# Comparative paleovirological analysis of crustaceans identifies multiple widespread viral groups

**DOI:** 10.1186/s13100-015-0047-3

**Published:** 2015-09-16

**Authors:** Gabriel Metegnier, Thomas Becking, Mohamed Amine Chebbi, Isabelle Giraud, Bouziane Moumen, Sarah Schaack, Richard Cordaux, Clément Gilbert

**Affiliations:** Université de Poitiers, UMR CNRS 7267 Ecologie et Biologie des Interactions, Equipe Ecologie Evolution Symbiose, building B8-B35, 6 rue Michel Brunet, TSA 51106 F-86073 Poitiers, Cedex 9 France; Department of Biology, Reed College, Portland, OR USA

**Keywords:** Paleovirology, Endogenous viral elements, Virus, *Bunyaviridae*, *Circoviridae*, *Mononegavirales*, *Parvoviridae*, *Totiviridae*, Copepoda, Crustacea

## Abstract

**Background:**

The discovery of many fragments of viral genomes integrated in the genome of their eukaryotic host (endogenous viral elements; EVEs) has recently opened new avenues to further our understanding of viral evolution and of host-virus interactions. Here, we report the results of a comprehensive screen for EVEs in crustaceans. Following up on the recent discovery of EVEs in the terrestrial isopod, *Armadillidium vulgare*, we scanned the genomes of six crustacean species: a terrestrial isopod (*Armadillidium nasatum*), two water fleas (*Daphnia pulex* and *D. pulicaria*), two copepods (the salmon louse, *Lepeophtheirus salmonis* and *Eurytemora affinis*), and a freshwater amphipod (*Hyalella azteca*).

**Results:**

In total, we found 210 EVEs representing 14 different lineages belonging to five different viral groups that are present in two to five species: *Bunyaviridae* (−ssRNA), *Circoviridae* (ssDNA), *Mononegavirales* (−ssRNA), *Parvoviridae* (ssDNA) and *Totiviridae* (dsRNA). The identification of shared orthologous insertions between *A. nasatum* and *A. vulgare* indicates that EVEs have been maintained over several millions of years, although we did not find any evidence supporting exaptation. Overall, the different degrees of EVE degradation (from none to >10 nonsense mutations) suggest that endogenization has been recurrent during the evolution of the various crustacean taxa. Our study is the first to report EVEs in *D. pulicaria*, *E. affinis* and *H. azteca*, many of which are likely to result from recent endogenization of currently circulating viruses.

**Conclusions:**

In conclusion, we have unearthed a large diversity of EVEs from crustacean genomes, and shown that four of the five viral groups we uncovered (*Bunyaviridae*, *Circoviridae*, *Mononegavirales*, *Parvoviridae*) were and may still be present in three to four highly divergent crustacean taxa. In addition, the discovery of recent EVEs offers an interesting opportunity to characterize new exogenous viruses currently circulating in economically or ecologically important copepod species.

**Electronic supplementary material:**

The online version of this article (doi:10.1186/s13100-015-0047-3) contains supplementary material, which is available to authorized users.

## Background

Our perception of viruses has shifted drastically during the last ten years owing to the rapid development of viral metagenomics methods [1]. Sequencing viral metagenomes from various environments has revealed that viruses are the most numerous and diverse organisms on Earth [2–4] and that, likely, only a small proportion of them are harmful pathogens. The results of these studies, coupled with the finding of many “good viruses”, suggest viruses could now often be considered mutualistic symbionts, fully integrated in holobionts, which have been defined as organisms harboring and interacting with a diverse microbial community [5, 6]. Viruses are thought to be at least as old as cellular organisms and it is becoming increasingly clear that they have had a strong, long-lasting, and ongoing influence on the evolution of their hosts and on ecosystem function [7–10].

The recent discovery that many viral genomes integrate into the genome of their eukaryotic hosts has shed new light on our understanding of viral evolution and on the evolution of host-virus interactions [11, 12]. Paleovirology, the study of these endogenous viral elements (EVEs), has produced several major breakthroughs. First, we have learned that many extant viral families are much older than what was previously thought and that fast rates of evolution inferred from currently circulating viruses cannot be extrapolated over long evolutionary periods of time [12]. Other interesting outcomes of paleovirology studies include, much like viral metagenomics, the dramatic expansion of the host ranges for viral families. For example, in a recent study, we performed a comprehensive bioinformatic screen for EVEs in the genome of a terrestrial crustacean isopod, the pill bug *Armadillidium vulgare* [13]. We uncovered 54 EVEs from 10 diverse lineages belonging to the *Bunyaviridae*, *Circoviridae*, *Parvoviridae* and *Totiviridae* families as well as to the *Mononegavirales* order, indicating that isopods have been and may still be exposed to a remarkable diversity of viruses. These findings extended the host range of all five viral groups to isopod crustaceans, and led to the question of whether *A. vulgare* is unique in terms of abundance and diversity of EVEs among crustaceans or if a diverse EVE biota is characteristic of the group as a whole. In order to address this question, and to shed new light on the dynamics of viral endogenization more generally, we extended our screen to another species of terrestrial isopod (*A. nasatum*) and to five additional crustacean species (two species of water flea [*Daphnia pulex* and *Daphnia pulicaria*]*,* a marine copepod [*Eurytemora affinis*], a freshwater amphipod [*Hyalella azteca*]*,* and the salmon louse [*Lepeophtheirus salmonis*; Copepoda]; Additional file 1: Figure S1)*.*

## Results

### EVE abundance and diversity in crustacean genomes

**Fig. 1:**
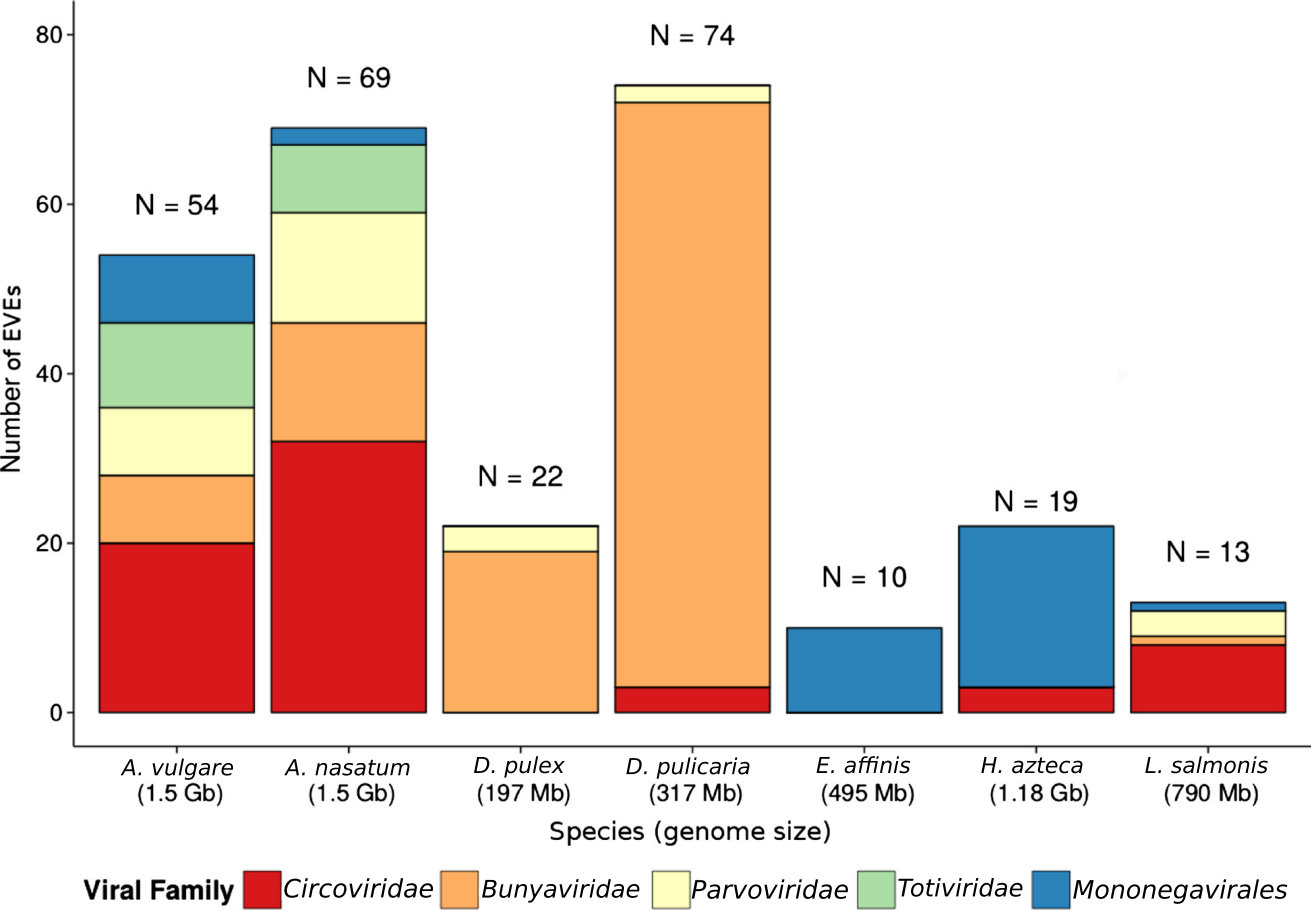
Numbers of endogenous viral elements from each viral group in the six crustacean species screened in this study. The size of their respective genomes is written below the species names. EVE numbers for *A. vulgare* are taken from Thézé et al. [13]. It is noteworthy that several EVEs share the same putative flanking region within a given species (see Results section and Additional file 3: Table S2), indicating that they were likely generated by post-insertional duplication (seven such events in total). The total number of endogenization events producing the 210 EVEs identified in this study is therefore lower than 210

**Fig. 2:**
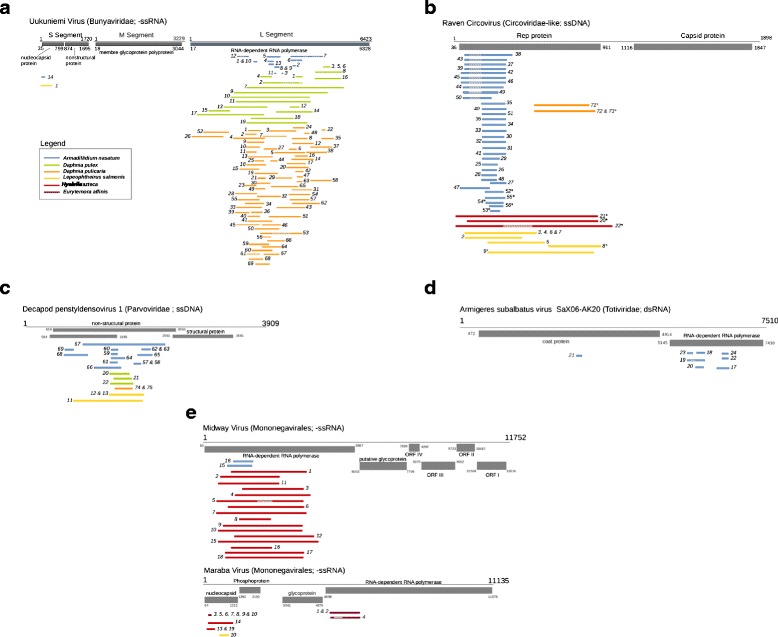
Schematic alignment of the 210 crustacean EVEs discovered in this study aligned to representative virus genomes belonging to **a** *Bunyaviridae* (Uukuniemi virus : Segment S, NC005221; Segment M, NC005220; Segment L, NC005214), **b** *Circoviridae* (Raven Circovirus : NC008375; EVEs followed by an « * » are schematically aligned on Dragonfly Orbiculatus virus [NC_023854] due to their low similarity to the Raven Circovirus), **c** *Parvoviridae* (Decapod penstyldensovirus 1 : NC002190), **d** *Totiviridae* (*Armigeres subalbatus* virus SaX06-AK20 : NC014609) and **e** *Mononegavirales* (Midway virus: NC012702 and Maraba virus : NC025255). Virus genes are represented in gray, with the coordinates of their Open Reading Frames below. Numbered and colored lines represent EVEs. Portions of EVEs with slanted black lines on white background are very divergent from reference virus sequences (in *Armadillidium nasatum* EVEs 5–7, 12, 19, 37–39, 42–46, 49, 50; *Daphnia pulex* 2; *D. pulicaria* 53; *Hyalella azteca* 5 and 22 and *Eurytemora affinis* 4)

Overall, our comprehensive screening for EVEs in six crustacean genomes led to the discovery of a total of 210 EVEs belonging to five viral groups (*Bunya*-, *Circo*-, *Parvo*-, *Toti*-*viridae* and *Mononegavirales*; Figs. 1 and 2). All EVEs are provided in Additional file 2: Dataset S1. This search revealed 69 EVEs in *A. nasatum*, 22 in *D. pulex* (most of which correspond to the phlebovirus-like EVEs reported by Ballinger et al. [14]), 74 in *D. pulicaria*, 10 in *E. affinis*, 22 in *H. azteca* and 13 in *L. salmonis* (Fig. 1). Among these 210 EVEs, 103 showed the highest amino acid identity to members of the *Bunyaviridae* (best blastp hits range from 24 to 73 % identities; average length = 242 aa), 46 were most similar to members of the *Circoviridae* (best blastp hits are 29 to 74 % identities; average length = 128 aa), 32 to members of the *Mononegavirales* (25 to 51 % identities; average length = 745 aa), 21 to members of *Parvoviridae* (best blastp hits are 28 to 100 % identities; average length = 118 aa) and 8 to *Totiviridae* (best blastp hits are 27 to 49 % identities; average length = 126 aa) (Additional file 3: Table S2).

Several lines of evidence indicate that the viral genome fragments detected in this study are integrated in the genome of their host, rather than circulating as free viruses. First, assuming that exogenous viruses were sequenced and assembled together with the targeted crustacean genomes, we should have been able to uncover entire viral genomes. Yet, our search only revealed pieces of viral genomes (Fig. 2). Secondly, the method used to sequence the six crustacean genomes did not involve a reverse transcription step, and thus did not allow sequencing of any RNA molecule. Yet, many of the EVEs we found originate from exogenous RNA viruses (*Bunyaviriridae*, *Mononegavirales* and *Totiviridae*). Thirdly, the presence of all 12 EVEs we targeted in isopods was confirmed by PCR amplification and Sanger sequencing (see below).

The number of EVEs detected in the various crustacean species varies substantially, from 10 in the copepod *E. affinis* to 74 in the water flea *D. pulicaria*. Though these differences may have biological underpinnings, they may also be in part explained by the varying quality of the genome assemblies (suggested by Geering et al. [15] and Zhuo et al. [16]), which varies greatly between species (Additional file 4: Table S1). Regarding the mechanisms underlying integration of viral genomes into crustacean genomes, we could not detect any sequence signature indicative of transposition-mediated or microhomology-mediated insertion. However, we found that several EVEs share the same putative flanking region within a given species (Additional file 3: Table S2), indicating that they were likely generated by post-insertional duplication (one such duplication in *E. affinis*, in *D. pulex*, in *H. azteca* and in *L. salmonis*; three such events in *D. pulicaria*; Additional file 3: Table S2).

### Phylogenies of crustacean endogenous viral elements

To better characterize the diversity of crustacean EVEs uncovered in this study and to decipher their evolutionary history, we aligned these EVEs with several representative exogenous (and sometimes endogenous) viruses for each family, including the *A. vulgare* EVEs described in Thézé et al. [13], and carried out phylogenetic analyses. All resulting trees are overall congruent with the trees described by the International Committee on Taxonomy of Viruses [17].

### *Bunyaviridae*

**Fig. 3:**
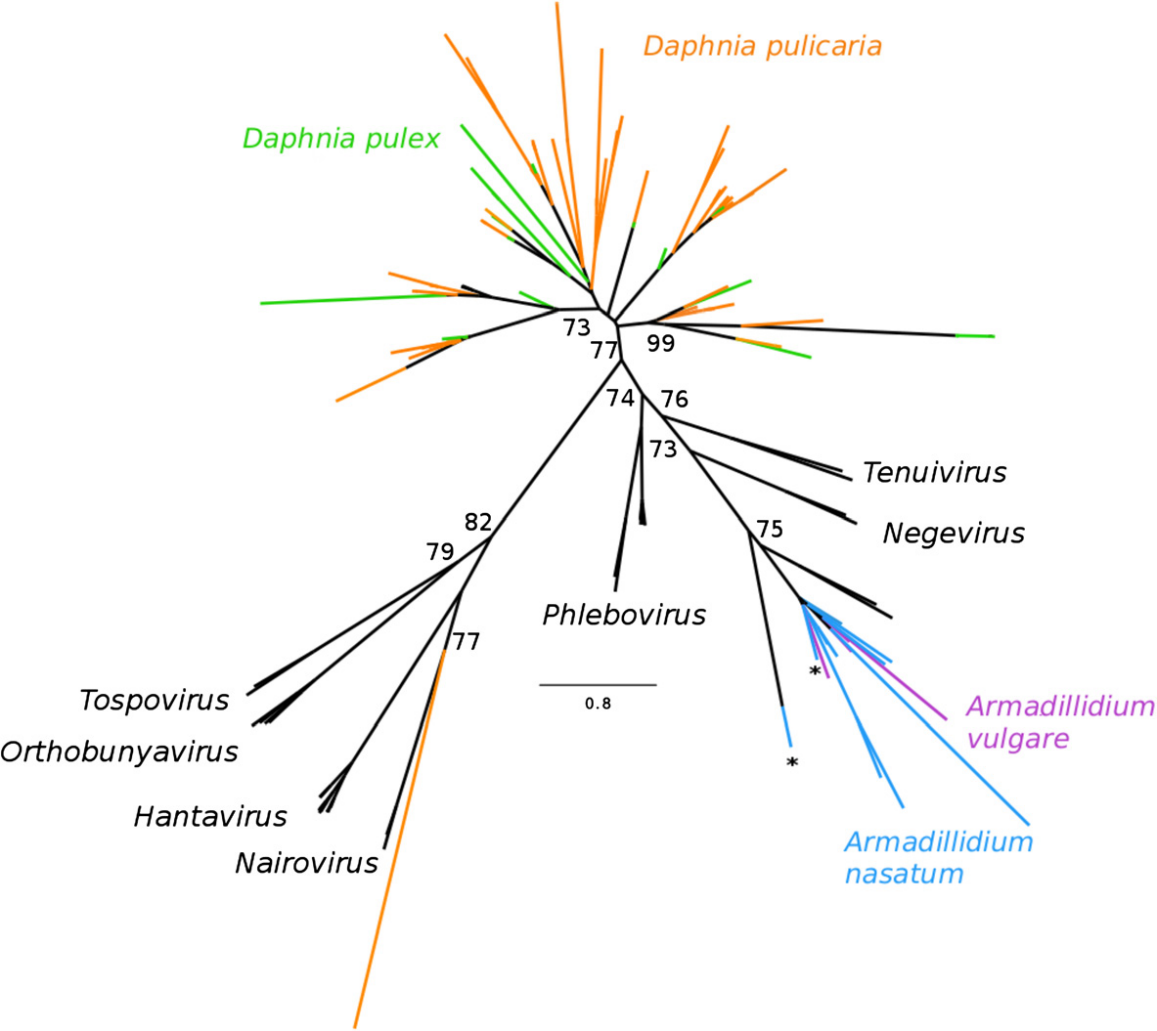
Phylogeny of the *Bunyaviridae* family, based on a multiple amino acid alignment and ML analysis of the RdRp. In addition to the EVEs discovered in this study, we added sequences from endogenous or exogenous viruses from the *Bunyaviridae* family. ML nonparametric bootstrap values (100 replicates) are indicated when > 70

In the RNA-dependent RNA polymerase (RdRp) phylogeny (Fig. 3), the 12 *A. nasatum* (sequences 1–11 and 13) *Bunyaviridae*-like EVEs are all closely related to *A. vulgare Bunyaviridae*-like EVEs described in Thézé et al. [13], forming a relatively well-supported cluster with recently described unclassified exogenous ssRNA viruses infecting arthropods [18]. *Daphnia Bunyaviridae*-like EVEs fall into two distinct lineages: one that includes all *D. pulex* and all but one *D. pulicaria* sequences, which is not closely related to any previously known Bunyavirus, and one corresponding to a single *D. pulicaria* sequence (*D. pulicaria* 48) that is related to the Nairovirus genus.

In the nucleocapsid phylogeny (Additional file 5: Figure S2), the *A. nasatum* sequence (*A. nasatum* 12) belongs to the same lineage as the *A. vulgare* sequence reported by Thézé et al. [13]. Given the global differences in the topology of the RdRp and nucleocapsid phylogenies, we cannot conclude whether the RdRp and nucleocapsid EVEs found in isopods originate from the same virus (or same viral lineage) or not. In the discussion, we conservatively assume that they come from the same exogenous virus. Finally, the *L. salmonis* nucleocapsid EVE fragment (*L. salmonis* 1) falls near Orthobunyaviruses and the unclassified Wuhan Fly ssRNA virus [18] but we cannot determine if this sequence belongs to one of the lineages described on the RdRp phylogeny.

### *Mononegavirales*

**Fig. 4:**
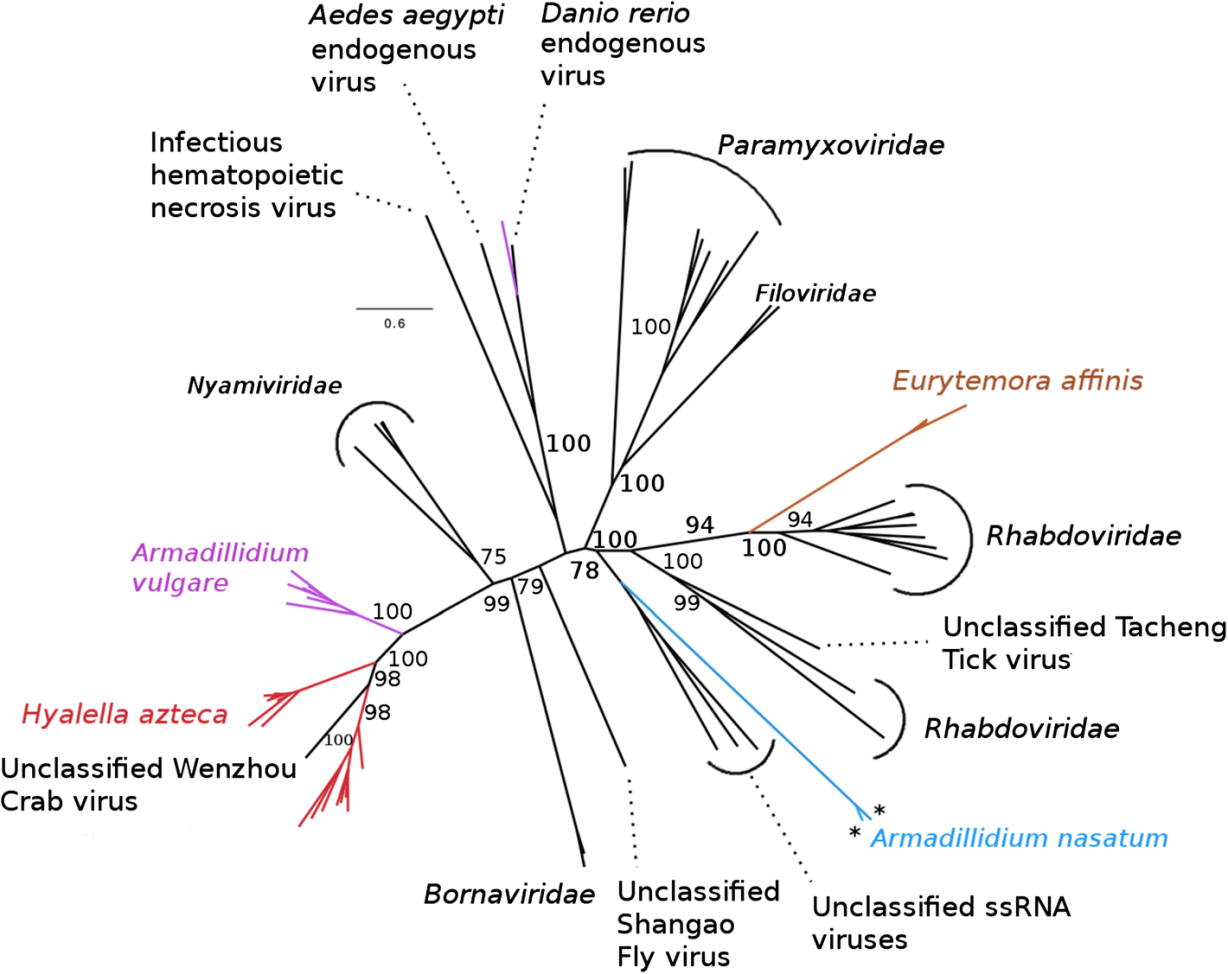
Phylogeny of the *Mononegavirales* group, based on a multiple amino acid alignment and ML analysis of the *Mononegavirales*-like RdRp. In addition to the EVEs discovered in this study, we added sequences of endogenous or exogenous viruses from the *Mononegavirales* group. ML nonparametric bootstrap values (100 replicates) are indicated when > 70

In the *Mononegavirales* RdRp phylogeny (Fig. 4), the newly described crustacean EVEs fall into three distinct lineages (without considering *A. vulgare* EVEs reported in Thézé et al. [13]). The first one includes the *H. azteca* EVEs, which cluster with the recently described unclassified exogenous Wenzhou crab virus [18] (bootstrap value = 100) and one of the *A. vulgare* EVE lineages described in Thézé et al. [13]. The second lineage corresponds to the two *A. nasatum* EVEs which group with unclassified ssRNA exogenous viruses infecting arthropods reported by Li et al. [18]. The third new lineage of crustacean *Mononegavirales*-like EVEs groups the two sequences from *E. affinis* which fall within the *Rhabdoviridae* family (bootstrap value = 100). In the nucleocapsid phylogeny (Additional file 6: Figure S3), the EVEs newly discovered in *E. affinis*, *L. salmonis* and *H. azteca* fall within the *Rhabdoviridae* family of exogenous viruses.

Given their similar placement in the two trees, it is likely that the *E. affinis* RdRp and nucleocapsid EVEs originate from the same *Rhabdoviridae*-like exogenous virus (or viral lineage). Interestingly, the placement of the *H. azteca* nucleocapsid EVEs (within *Rhabdoviridae*; Additional file 6: Figure S3) clearly differs from that of the RdRp EVEs found in this species (distantly related to *Rhabdoviridae*; Fig. 4), suggesting that the two types of sequences originated from two distinct viral lineages. It is also noteworthy that the *L. salmonis* sequence clusters tightly with the two sequences of exogenous Rhabdoviruses reported in this same host species by Okland et al. [19] (bootstrap value = 80), suggesting that it must result from a relatively recent endogenization event.

### *Circoviridae*

**Fig. 5:**
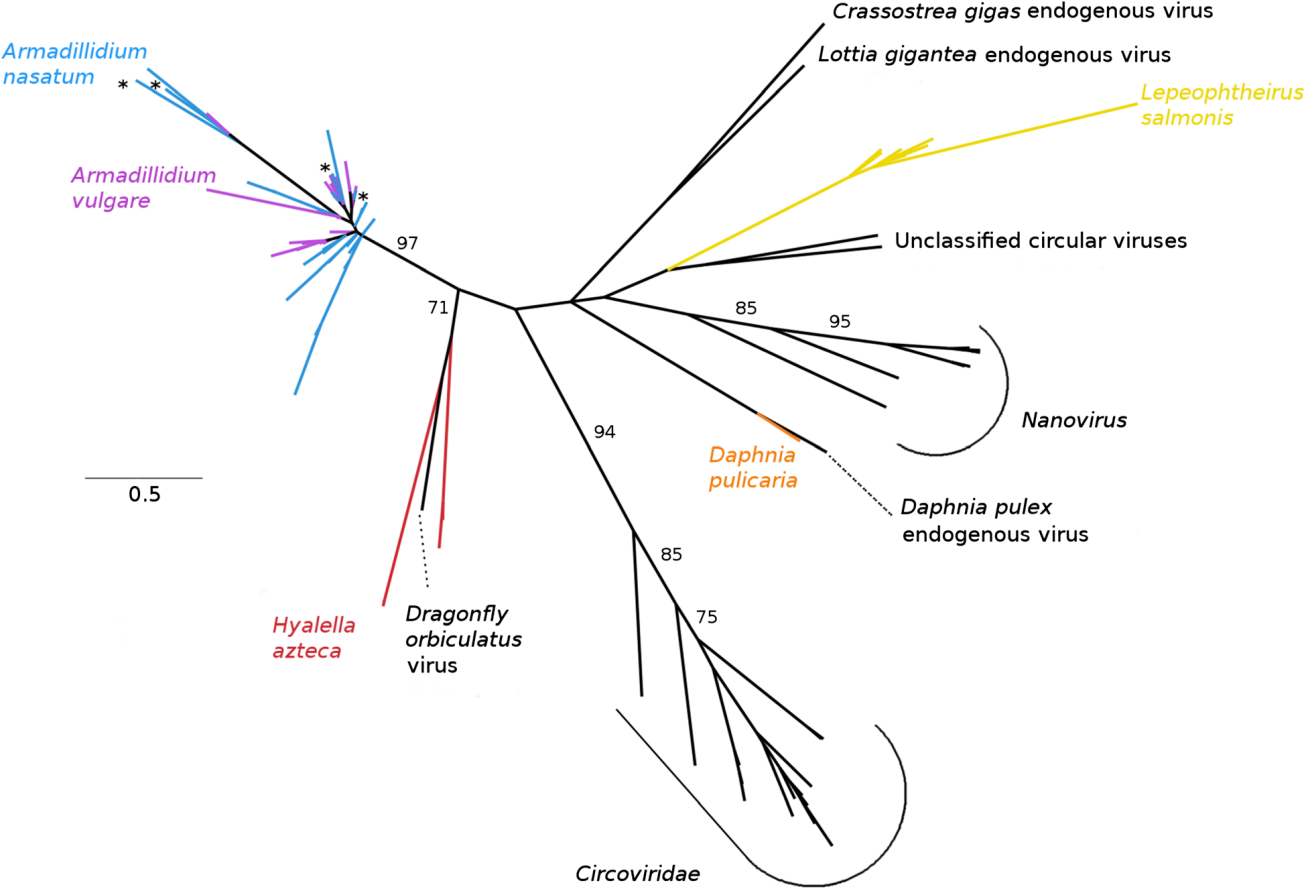
Phylogeny of the *Circoviridae* family, based on a multiple amino acid alignment and ML analysis of the *Circoviridae*-like rep protein. In addition to the EVEs discovered in this study, we added sequences of endogenous or exogenous viruses from the *Circoviridae* family. ML nonparametric bootstrap values (100 replicates) are indicated when > 70

In the *Circoviridae* phylogeny (Fig. 5), crustacean EVEs fall into three distinct lineages: (1) corresponding to the *A. nasatum* EVEs and *A. vulgare* EVEs (described in Thézé et al. [13]), (2) a large cluster including *L. salmonis* and *Daphnia* EVEs together with Nanoviruses, unclassified exogenous circovirus-like sequences obtained from environmental metagenomics [20, 21], and endogenous viruses from mollusks (included in the Thézé et al. [13] phylogeny), and (3) a group linking *H. azteca* EVEs to the Dragonfly orbiculatus exogenous virus reported in Rosario et al. [22].

### *Parvoviridae*

**Fig. 6:**
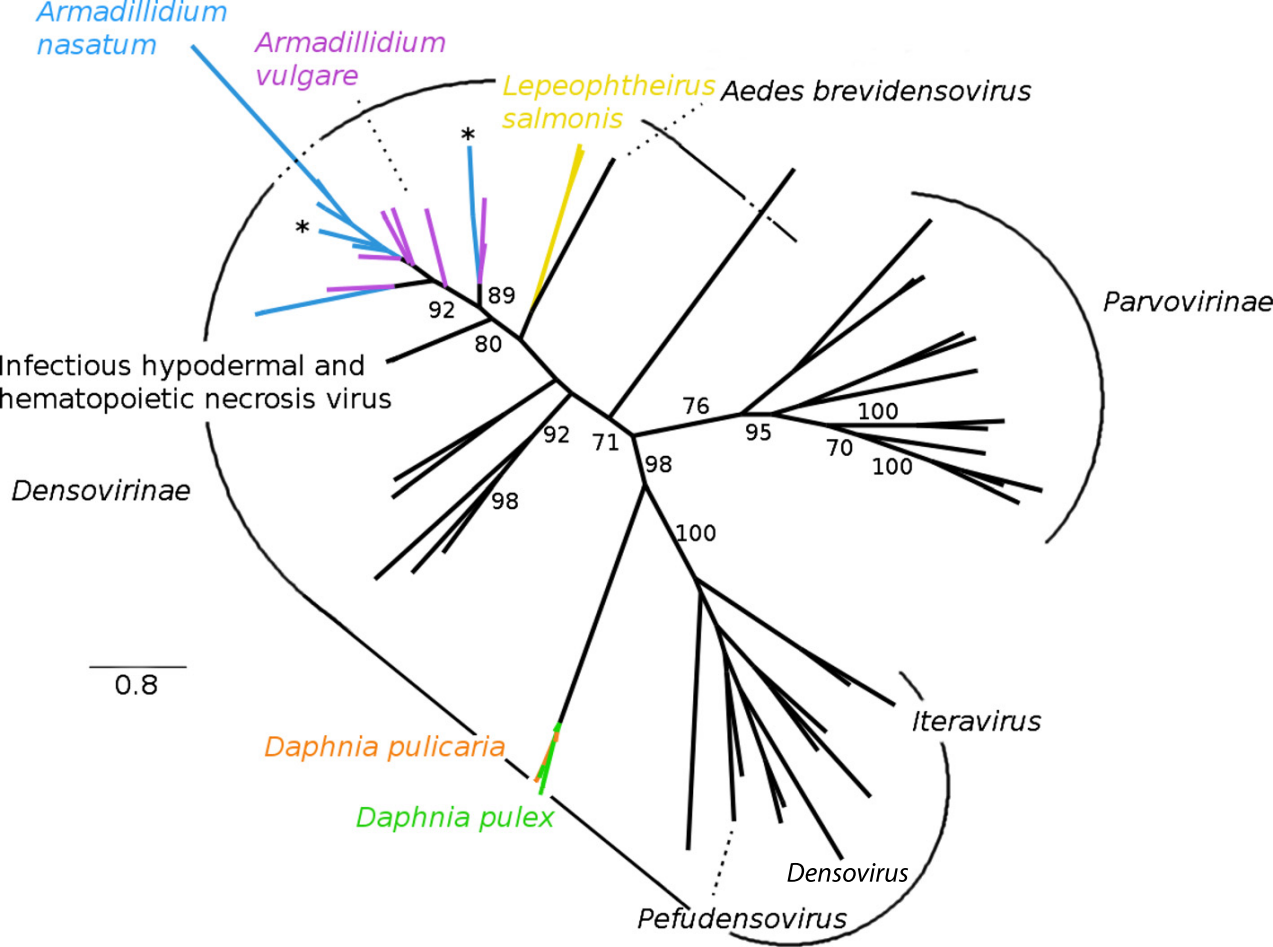
Phylogeny of the *Parvoviridae* family, based on a multiple amino acid alignment and ML analysis of the *Parvoviridae*-like non-structural protein. In addition to the EVEs discovered in this study, we added sequences of exogenous viruses from the *Parvoviridae* family. ML nonparametric bootstrap values (100 replicates) are indicated when > 70

Crustacean *Parvoviridae*-like EVEs fall into at least two distinct lineages within the Densovirinae (Fig. 6). The first one corresponds to *Daphnia* EVEs and is related to the Densovirus, Pefudensovirus and Iteravirus genera. The second one includes *Armadillidium* and *L. salmonis* EVEs, as well as exogenous Brevidensoviruses from *Aedes* mosquitoes [23, 24], and the exogenous and endogenous versions of the Infectious Hypodermal and Hematopoietic Necrosis Virus [25, 26]. Though *L. salmonis* EVEs seem more closely related to mosquito brevidensoviruses than to *Armadillidium* EVEs, we conservatively assume that all these sequences are part of the same large lineage (bootstrap value = 80) because there is no large phylogenetic gap between them, *i.e.*, the distance separating each branch are relatively homogeneous.

### *Totiviridae*

**Fig. 7:**
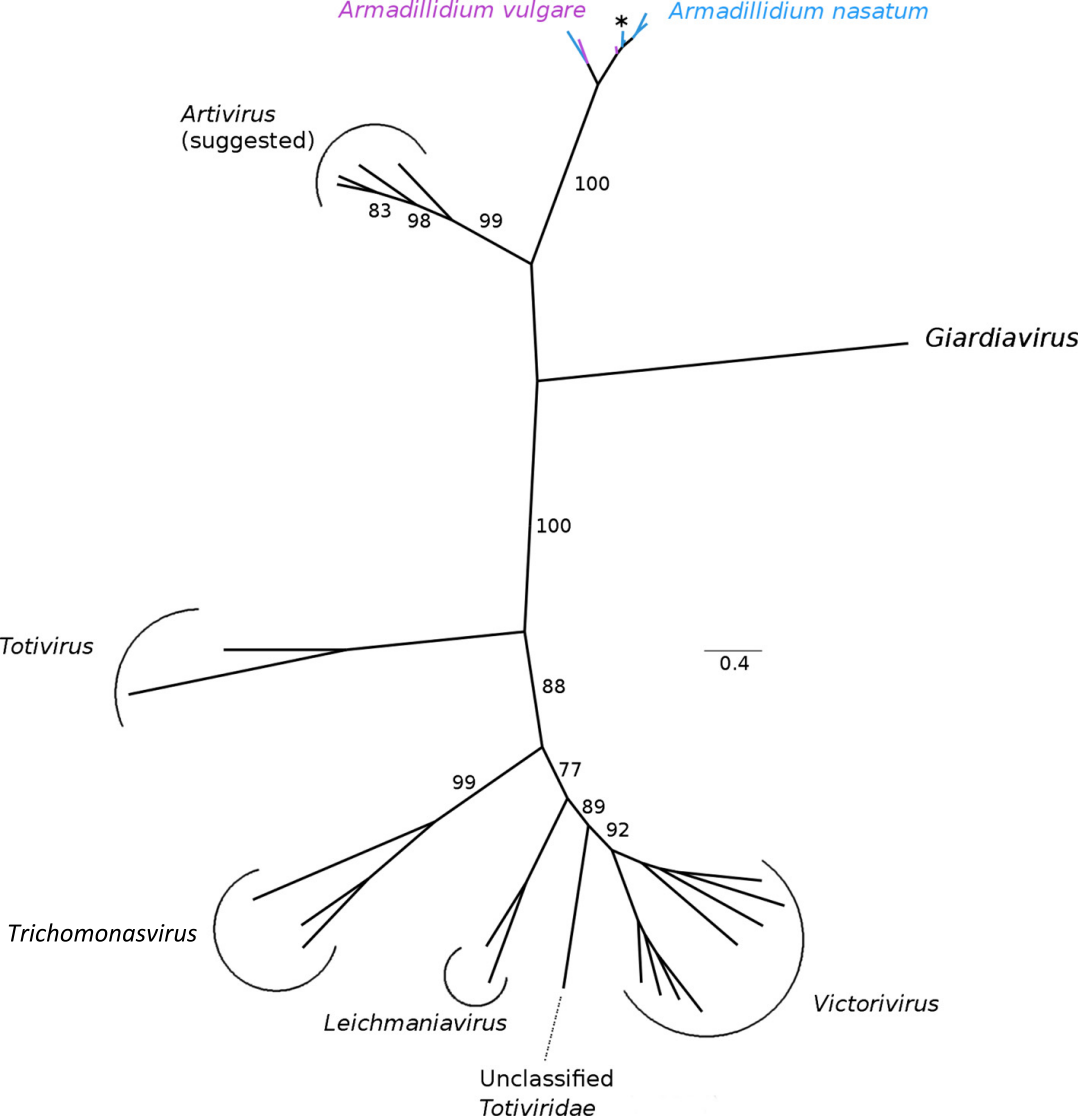
Phylogeny of the *Totiviridae* family, based on a multiple amino acid alignment and ML analysis of the RdRp. In addition to the EVEs discovered in this study, we added sequences of exogenous virus viruses from the *Totiviridae* family. ML nonparametric bootstrap values (100 replicates) are indicated when > 70

The only *Totiviridae*-like EVEs we found were in *A. nasatum*. Both RdRp and coat protein fragments (Fig. 7 and Additional file 7: Figure S4) were found in this species, and they cluster with the lineage of *A. vulgare Totiviridae*-like EVEs described in Thézé et al. [13]. This is most closely related to exogenous viruses from the Artivirus genus and to a virus from the unicellular eukaryote Giardia [13, 27–30].

### Orthologous endogenous viral elements

We obtained positive PCR products for all 12 EVEs screened using the genomic DNA sample that served for sequencing the *A. nasatum* genome. Most of these 12 EVE loci were also amplified and Sanger sequenced in the other two *A. nasatum* DNA samples (Table 1), except the *Bunyaviridae*-like EVE 7 (negative PCR in *A. nasatum* sample 2 [An2]), *Mononegavirales*-like EVEs 15 and 16 (negative PCR in An2 and An3), and the *Parvoviridae*-like EVE 67 (negative PCR in An3). Our *in silico* search for orthologous EVEs revealed three loci shared between *A. vulgare* and *A. nasatum* for which the host origin of the flanking region could be identified unambiguously (Additional file 8: Figure S5): *A. nasatum* Bunyavirus-like EVE 12, *A. nasatum* Circovirus-like 50 and *A. nasatum* Circovirus-like EVE 44. Not only are the flanking regions of these loci not similar to any known viral sequence, but they are characterized by the presence of interspersed and/or long microsatellite repeats (repeated at least six times or more). Such repeats are absent from the genome of all viruses belonging to the *Circoviridae* and *Bunyaviridae*, indicating that what we have identified as flanking regions indeed correspond to the eukaryotic host (*Armadillidium*) rather than the viral genome (Additional file 8: Figure S5). In addition to the sequences obtained from the genome sequence of *A. nasatum* and *A. vulgare*, we were able to PCR/Sanger sequence the three loci in *A. tunisiense* and one of them in *A. depressum* (Additional file 8: Figure S5). All EVEs identified computationally in *A. nasatum* or using PCR/sequencing were deposited in Genbank under accession numbers KT713978 – KT714035.

**Table 1:** EVEs PCR amplifications in 4 species of terrestrial isopods

Viral Family	EVE^a^	An1	An2	An3	At	Ad	Av
*Bunyaviridae*	7	+	-	+	-	-	-
*Bunyaviridae*	12	+	+	+	+	+	-
*Mononegavirales*	15	+	-	-	-	-	-
*Mononegavirales*	16	+	-	-	-	-	-
*Totiviridae*	21	+	+	+	-	-	+
*Totiviridae*	23	+	+	+	-	-	-
*Circoviridae*	44	+	+	+	+	-	-
*Circoviridae*	45	+	+	+	-	-	-
*Circoviridae*	46	+	+	+	-	-	+
*Circoviridae*	50	+	+	+	+		-
*Parvoviridae*	67	+	+	-	+	+	-
*Parvoviridae*	69	+	+	+	-	-	-

An: *Armadillidium nasatum* (three individuals were screened); At : *A. tunisiense*; Ad : *A. depressum*; Av : *A. vulgare*

+: Amplified; - : Not amplified

^a^Each EVE is identified by a number as in Fig. 2

## Discussion

Until recently, most of the knowledge available on crustacean viruses derived from studies of disease-causing viruses in shrimp farming, such as the white spot syndrome virus (WSSV; *Nimaviridae*), the taura syndrome tirus (TSV; *Picornaviridae*), and the yellowhead virus (YHV; *Roniviridae*) [31, 32], as well as the infectious hypodermal and hematopoietic virus (IHHN; *Parvoviridae*) [33] and the infectious myonecrosis virus (IMNV; *Totiviridae*) [28]. In addition, invertebrate iridescent viruses (*Iridoviridae*; dsDNA) have been observed in four species of decapods, two species of maxillopods, two species of branchiopods and 18 species of isopods [34]. These viruses are relatively easy to detect because of the iridescent blue or red color of infected individuals [35–37]. Dunlap et al. [38] also described a circovirus infecting two ecologically important copepod species, and two new species of rhabdoviruses were recently characterized in the salmon louse [19].

We recently discovered EVEs in *Armadillidium vulgare* and showed that terrestrial crustacean isopods have been and may still be exposed to a large variety of viruses, many of which belong to viral lineages that had never been reported in crustaceans before [13]. Here, we show that members of all five viral groups found in *A. vulgare* (*Bunyaviridae*, *Circoviridae*, *Mononegavirales*, *Parvoviridae*, *Totiviridae*) have also become endogenized in another terrestrial isopod, *A. nasatum,* and that four other crustacean species each harbor a viral flora composed of a subset of these five viral groups as well. Interestingly, all but one EVE lineage found in *A. nasatum* group with those previously identified in *A. vulgare*, which suggest that the two species are infected by the same viruses, an observation which is consistent with the fact that the distribution of the two species largely overlaps in Europe and that they are often found in the same habitats [39]. Overall, our phylogenetic analyses revealed that crustacean EVEs tend to group by taxa in distinct, well supported clusters across no fewer than 14 distinct viral lineages: four *Bunyaviridae*, five *Mononegavirales* (including a new *Armadillidium* lineage in *A. nasatum*), two *Circoviridae*, two *Parvoviridae* and one *Totiviridae*, 10 of which were also found in the initial screen of *A. vulgare* [13].

Given the tremendously large diversity of viruses known to infect eukaryotes and the fact that we screened species that are widely divergent form each other and from *A. vulgare*, it is perhaps surprising that all new EVEs detected here belong to the same viral groups than those detected in *A. vulgare* (no additional viral family was detected) and that only four of the lineages reported here were not found in *A. vulgare*. This leads to three non-mutually exclusive hypotheses: (1) that these five viral groups are simply the most widespread in crustaceans, (2) these viral groups are more likely to endogenize than other viruses without being more prevalent as exogenous viruses, or (3) that crustacean genomes are uniquely vulnerable to endogenization by these 5 groups, relative to other host genomes. We note that a member of at least one other viral family (*Nimaviridae*) has been unearthed from a crustacean genome [40], and we believe that as more metagenomics and paleovirology studies are conducted, comparing global patterns of endogenization and global viral flora of extant viruses in a given taxonomic group will yield interesting insights into the ecology of host/virus interactions. But our current knowledge in this area is still too limited to draw any firm conclusion on this aspect of our results.

Our alignment of crustacean EVEs to representative exogenous viruses from each of the five viral groups revealed that most EVE fragments (83 %) are from the polymerase, with the remaining fragments being derived from different open reading frames such as coat or nucleocapsid protein (Fig. 2). Because this pattern is consistent throughout all five viral groups, we believe it is most likely explained by the strong purifying selection pressures acting on viral polymerases [41–44], leading to a high degree of conservation of such proteins between viruses that were endogenized and extant exogenous viruses. The other structural proteins (coat or capsid proteins) tend to be involved in more direct interactions with host factors (such as cell receptors) and are key to the entry of the virus in the cell. Thus, they are more likely to be engaged in an evolutionary arms race with the host and to evolve under rapid positive selection (e.g. [45, 46]). The level of similarity of such proteins between endogenized viruses and extant ones is therefore expected to be lower than that observed for polymerases.

The crustacean EVEs detected in this study show various levels of degradation when compared to their closest exogenous virus relatives, some being intact or disrupted by just one or a few mutations inducing a stop codon and/or a frameshift and others being heavily degraded by more than 10 nonsense mutations (Additional file 3: Table S2). This pattern indicates that viral endogenization has been recurrent during the evolution of the taxa included in this study. Further suggesting recurrent endogenization over time, we identified three EVEs shared at orthologous loci between *A. nasatum* and two or three other *Armadillidium* species (Additional file 8: Figure S5), and we were unable to amplify three other EVEs by PCR in one or two *A. nasatum* individuals sampled from a different population than the one used for genome sequencing (Table 1). These data indicate that, while some EVEs are old and were endogenized before the split between *A. nasatum* and the other *Armadillidium* species, others are more recent and are likely to still be polymorphic (with respect to presence/absence patterns) in *A. nasatum*. The phylogenetic relationships of the three *Armadillidium* species included in our study have yet to be robustly resolved, but the mitochondrial COI gene from the two most distantly relatives (*A. nasatum* and *A. vulgare,* according to Dupeyron et al. [47]) differ by 16 %. Considering the proposed COI substitution rate of 1.4 % per million years in decapods [48], we can infer that the EVEs detected in isopods result from recurrent endogenization events that took place over several millions of years during the evolution of terrestrial isopods. The three EVEs that became endogenous in the ancestor of the *A. nasatum* + *A. vulgare* clade are all disrupted by one to four nonsense mutations and we did not find evidence for their transcription in the transcriptome of *A. nasatum* and *A. vulgare* [49]. Thus unlike previously described examples in non-crustacean taxa (e.g. [50, 51]), these three isopod EVEs do not appear to evolve under purifying selection and to fulfill a cellular function. Their maintenance in isopod genomes over several millions of years is therefore either completely neutral or due to initial exaptation, followed by loss of function and ongoing degradation as proposed for *Syncytin* genes in primates [52].

Finally, this study is the first to report viruses in the water flea *D. pulicaria*, the amphipod *H. azteca,* and the copepod *E. affinis.* The latter species is a major component of the mesozooplankton found in various saline and freshwater environments of the northern hemisphere [53]. Viruses have an important impact on the structure and ecology of phytoplankton communities [54], and it has recently been suggested they may play an important role in shaping mesozooplankton communities as well [38]. In addition, there is evidence suggesting that copepods can serve as vectors for transmitting viruses to fish and shrimp, causing important economic losses [55, 56], and to phytoplankton, with possible consequences on global biogeochemical cycling [57]. Despite these major consequences, only one study has characterized viral infections in copepods so far [38]. Interestingly, many of the copepod EVEs are devoid of nonsense mutation (Additional file 3: Table S2), suggesting they were endogenized very recently and may still be very similar at the nucleotide level to currently circulating viruses.

## Conclusions

In conclusion, we characterized a large diversity of EVEs in crustacean genomes resulting from recurrent events of endogenization taking place over several millions of years. Most EVEs correspond to non-structural viral proteins, likely reflecting the slower rate of change of these proteins as compared to structural proteins. Interestingly, we found that four viral groups (*Bunyaviridae*, *Circoviridae*, *Mononegavirales*, *Parvoviridae*) are widespread in crustaceans, being present in three to four highly divergent taxa (amphipods, copepods, isopods, branchiopods) and that all viral groups found in non-isopod crustaceans are present in isopods. We anticipate that further large scale paleovirology and metagenomics studies will shed light on the factors shaping global patterns of viral endogenizations and the composition of the viral communities currently circulating in a given taxonomic group. Finally, the sequences of recent EVEs that we identified in this study could facilitate the discovery of new exogenous viruses through targeted searches. The characterization of EVEs not only serves to provide a catalog of paleoviral events shedding light on past host-virus interactions but it can also help discovering new viruses in ecologically and/or economically important taxa (e.g. the copepods *E. affinis* and *L. salmonis*).

## Methods

### Genome screening for endogenous viral elements

The genomes of *E. affinis*, *H. azteca*, *L. salmonis* and *D. pulex* were downloaded from the GenBank database under accession numbers AZAI00000000, JQDR00000000, ADND00000000 and ACJG00000000 respectively. The genome of *D. pulicaria* was downloaded from the *wFleaBase* Internet repository. The whole genome sequences of *A. nasatum* used in this study were generated as part of the ongoing *A. nasatum* genome project in our laboratory. Briefly, total genomic DNA was extracted from two *A. nasatum* individuals. A paired-end library with ~230 bp inserts was prepared and sequenced on an Illumina HiSeq2000. Reads were filtered with FastQC and assembled using the SOAP de novo software version 2.04 [58]. The best assembly was obtained with a *k*-mer size of 61. Genome statistics are available for all species in Additional file 4: Table S1.

To search for endogenous viral elements in crustacean genomes, we first removed low complexity repeats from the six genomes using RepeatMasker 4.0.5 [59]. We then carried out tblastx similarity searches [60] on these genomes using all available viral genomes (n = 5678 as of April 2015) as queries. Crustacean sequences yielding tblastx hits were then parsed from the tblastx output and converted into a fasta file using a custom script. Many tblastx hits were false positives corresponding to repeated sequences or to eukaryote genes that are present in viruses following host-to-virus horizontal transfers, known to be common in large dsDNA viruses [13, 61–63]. In order to remove these false-positive sequences, a reciprocal blastp was carried out using the tblastx fasta output as query on the “nr” (non-redundant) Genbank protein database, to eliminate any sequences for which the best reciprocal blastp hit was not a virus. The remaining sequences were manually aligned to a reference viral genome in BioEdit 7.1.9 [64] in order to draw schematic maps to illustrate the viral genome fragments endogenized in crustacean genomes.

### Phylogenetic analyses

To better evaluate the diversity of newly discovered crustacean EVEs and to shed light on their evolutionary history, we carried out phylogenetic analyses of viral sequences including EVEs from Thézé et al. [13] and exogenous viral sequences obtained from Genbank. This phylogenetic analysis included closely related viral sequences selected following the BLAST analysis with the addition of closely-related proteins of representative virus species (International Committee on Taxonomy of Viruses, [17]), as well as recently published sequences [18] for two viral groups (*Bunyaviridae* and *Mononegavirales*). Triple iteration amino acid sequence multiple alignments were generated using ClustalOmega software (version 1.2.1; [65]). Maximum-Likelihood inferences were then performed on each alignment using the WAG empirical model of protein evolution [66] implemented in the RAxML software V. 7.4.6 [66]. Non-parametric bootstrap support values were obtained using parameters optimized for small datasets [67] after 100 iterations.

### PCR and *in silico* screening of orthologous crustaceans endogenous viral elements

We used PCR and Sanger sequencing to verify the presence of some of the EVEs identified computationally in the *A. nasatum* genome and to assess whether some of them are polymorphic in terms of presence/absence at orthologous genomic sites in *A. nasatum* individuals sampled from three different populations available in our laboratory. We also investigated whether these EVEs are present at orthologous sites in three other closely related isopod species (*A. depressum, A. tunisiense* and *A. vulgare*). For this analysis, we selected the two EVEs of each viral group (four for *Circoviridae* because we found many more EVEs for this viral group) with the longest flanking regions. We then designed PCR primers to the flanking regions (Additional file 9: Table S3) and conducted a series of PCR screens on three *A. nasatum* DNA samples, one *A. vulgare* sample, one *A. depressum* sample, and one *A. tunisiense* sample. Genomic DNA extraction followed the Wilson protocol [68] which involved 3 h incubation of the tissue sample in proteinase-K at 56 °C, centrifugation (8000 g, 2 min), and an RNAse treatment (30 min at 37 °C). DNA samples were then purified using spin columns from the DNeasy Blood & Tissue Kit (Qiagen). PCR reactions were carried out in 25 μl with 5 μL Buffer 5X, 0.5 μL dNTPs (2.15 mM), 1 μL of each primer (100 μM), 0.25 μL Taq polymerase 5 u/μL, 1 μL DNA. Thermocycling consisted of a 94 °C phase for 4 min, then 30 cycles of 30 s at 94 °C, 30 s at 55 °C and 50 s at 72 °C, followed by a final extension step of 5 min at 72 °C. PCR products resulting from amplifications in species other than *A. nasatum* were systematically purified and Sanger sequenced.

We also carried out an *in silico* screen to detect EVEs that are orthologous between *A. nasatum* and *A. vulgare*. For this we used all *A. nasatum* EVEs flanked on one or both sides by at least 150 bp of sequence showing no similarity to any virus as queries to perform blastn searches on *A. vulgare* sequences (using the sequences published in Thézé et al. [13]). Our identification of EVE flanking regions first relies on the fact that these regions are not similar to any known virus. In order to verify that they correspond to the host genome, we searched for the presence of known proteins motifs using these regions as queries to perform blastx searches against the Genbank non-redundant protein database. We also searched for the presence of interspersed repeats, which are typically abundant in eukaryotic genomes but very rare in viruses in general and absent from the genomes of *Bunyaviridae* and *Circoviridae* (the two viral families to which belong the three EVEs for which we found repeats in their flanking regions). For this we used each EVE flanking region as a query to perform blastn searches against the host genome it was extracted from. We considered interspersed repeats as regions longer than 100 bp repeated at least 10 times in the *Armadillidium nasatum* genome. The CENSOR searches we ran in Repbase [69] on the two interspersed repeats that we identified did not reveal any similarity to any known transposable element.

## Additional files

Additional file 1: Figure S1. Phylogenetic relationships of the species studied in this project. The species targeted are in red. Divergence times are from www.timetree.org, except for *Daphnia* [70]. (PDF 122 kb) Additional file 2: Dataset S1. Nucleotide sequence of all endogenous viral elements identified in this study. (TXT 201 kb) Additional file 3: Table S2. Characteristics of endogenous viral elements in six crustacean genomes. In the "PCR test" column, hyphens indicate that we did not attempt to amplify the locus by PCR, while the "✓" sign indicates that we successfully amplified the locus by PCR in *A. nasatum*. The EVEs sharing the same letter (A, B, C, D, E or F) in the "Post insertional duplications" column have identical flanking regions, suggesting they were generated by post insertional duplication. In the same column, hyphens indicate either no flanking region or no similarity of the flanking region to any other EVE locus. (ZIP 139 kb) Additional file 4: Table S1. Quality assessments of the 6 crustacean genomes used in this study. (DOCX 17 kb) Additional file 5: Figure S2. Phylogeny of the *Bunyaviridae* family, based on a multiple amino acid alignment and ML analysis of the nucleocapsid protein. In addition to the EVEs discovered in this study, we added sequences of exogenous viruses from the *Bunyaviridae* family. ML nonparametric bootstrap values (100 replicates) are indicated when > 70. (PDF 89 kb) Additional file 6: Figure S3. Phylogeny of the *Mononegavirales* group, based on a multiple amino acid alignment and ML analysis of the *Mononegavirales*-like nucleocapsid protein. In addition to the EVEs discovered in this study, we added sequences of exogenous viruses from the *Mononegavirales* group. ML nonparametric bootstrap values (100 replicates) are indicated when > 70. (PDF 92 kb) Additional file 7: Figure S4. Phylogeny of the *Totiviridae* family, based on a multiple amino acid alignment and ML analysis of the nucleocapsid protein. In addition to the EVEs discovered in this study, we added sequences of exogenous viruses from the *Totiviridae* family. ML nonparametric bootstrap values (100 replicates) are indicated when > 70. (PDF 74 kb) Additional file 8: Figure S5. Schematic representation of the three EVE loci that are orthologous between the various *Armadillidium* species. The plain green portion of the loci are similar to a virus. a) *A. nasatum* Bunyavirus-like EVE 12 is most similar to the Wuhan insect virus 1 RdRp (AJG39261). Its 3’ flank contains a 103-bp interspersed repeat (IR in blue) which is repeated at least 66 times in the *A. nasatum* genome (average similarity between repeats = 86 %) and a partial ORF similar to a hypothetical protein from *Helobdella robusta* (in grey). b) *A. nasatum* Circovirus-like EVE 44 is most similar to the Dragonfly orbiculatus virus rep protein (AFS65301). Its 5’ and 3’ flank contain a dinucleotide microsatellite (in orange) repeated at least 15 and 6 times respectively, that are shared at the exact same position with *A. vulgare*. c) *A. nasatum* Circovirus-like EVE 50 is most similar to the rep protein of an Uncultured marine virus (GAC77817).xIts 3’ flank contains a 130-bp interspersed repeat which is repeated at least 13 times in the *A. nasatum* genome (average similarity between repeats = 91 %), as well as a trinucleotide microsatellite repeated at least 17 times and shared with *A. vulgare*. The green portions of the loci with slanted black stripes correspond to the rest of the flanking regions, which are not similar to any known sequence. Red arrows indicate the position of forward and reverse PCR primers. (PDF 393 kb) Additional file 9: Table S3. PCR primers used to confirm the presence of EVEs uncovered by the bio-informatic analysis and to screen for orthologous insertions in 3 Armadillidae species : *Armadillidium vulgare*, *A. tunisiense* and *A. depressum.* (DOCX 17 kb)

## Abbreviations

RdRp: RNA-dependent RNA polymerase
PCR: Polymerase chain reaction
EVE: Endogenous viral element
