# Supplementary figures and images for: Comparative paleovirological analysis of crustaceans identifies multiple widespread viral groups
Source: Mob DNA. 2015 Sep 16;6:16. doi: 10.1186/s13100-015-0047-3 (PMC4573495; doi:10.1186/s13100-015-0047-3)

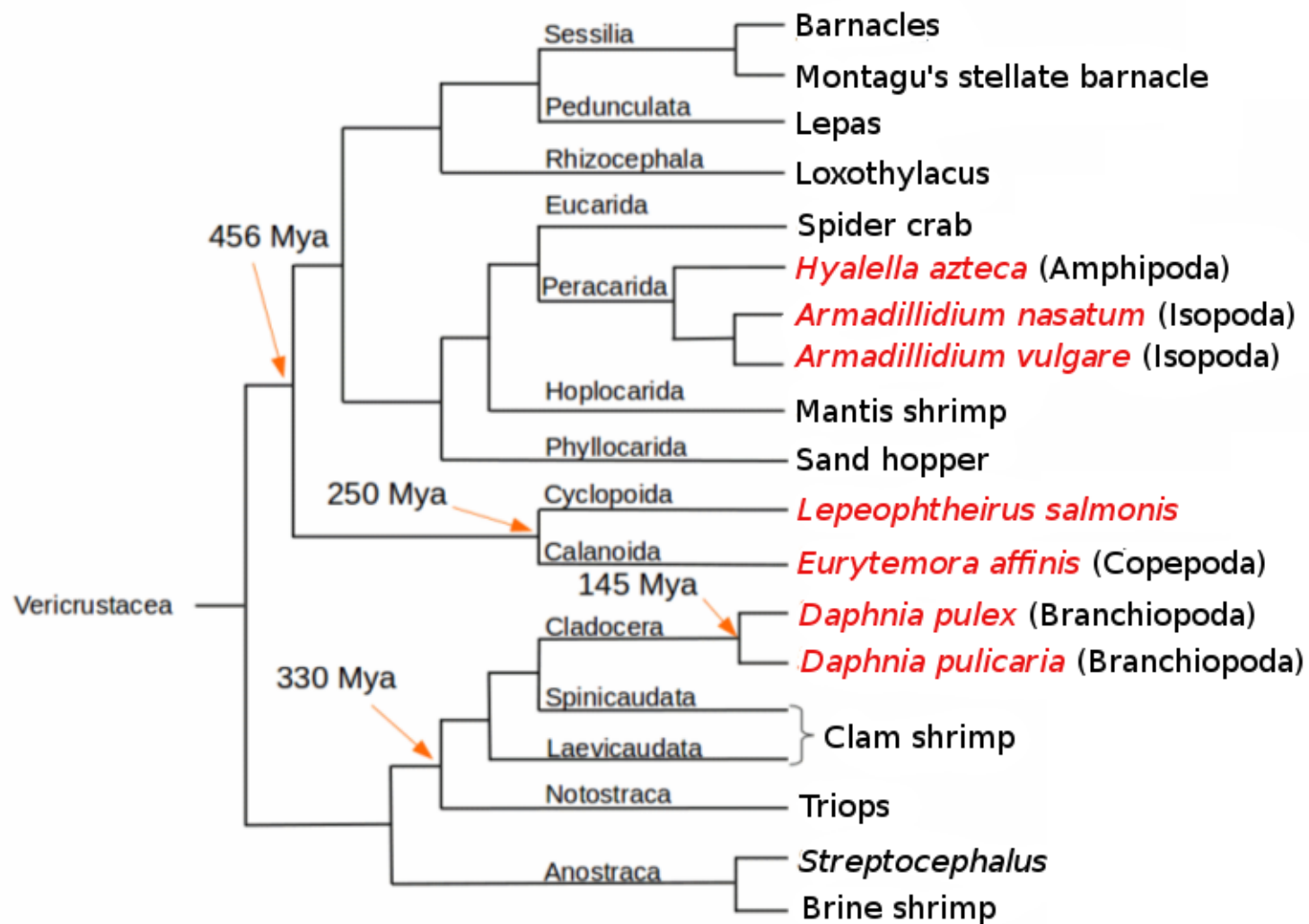

Fig. S1

Supplement: Additional file 1: Figure S1. — Phylogenetic relationships of the species studied in this project. The species targeted are in red. Divergence times are from www.timetree.org, except for Daphnia [70]. (PDF 122 kb) [file 13100_2015_47_MOESM1_ESM.pdf]

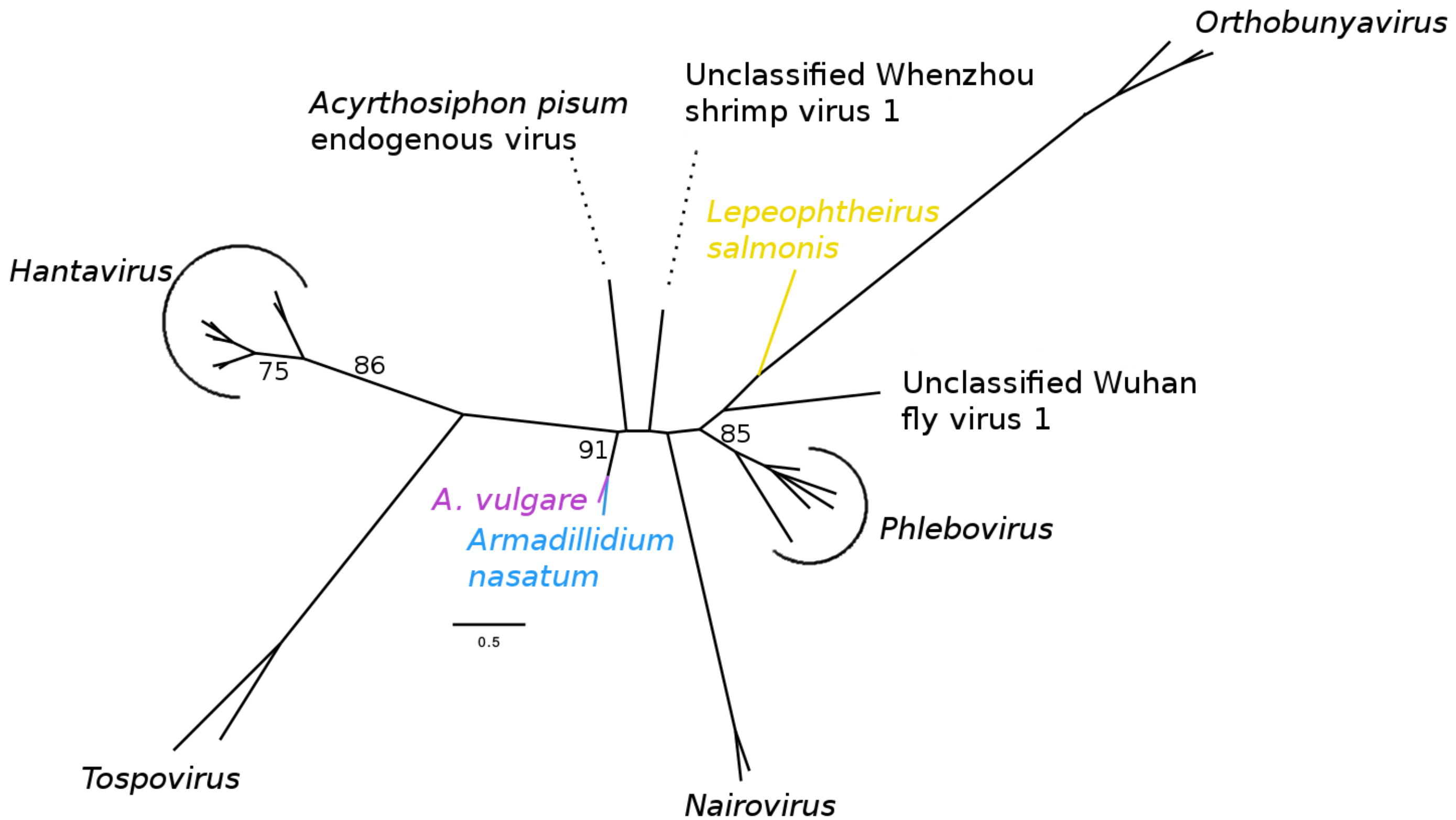

Fig. S2

Supplement: Additional file 5: Figure S2. — Phylogeny of the Bunyaviridae family, based on a multiple amino acid alignment and ML analysis of the nucleocapsid protein. In addition to the EVEs discovered in this study, we added sequences of exogenous viruses from the Bunyaviridae family. ML nonparametric bootstrap values (100 replicates) are indicated when > 70. (PDF 89 kb) [file 13100_2015_47_MOESM5_ESM.pdf]

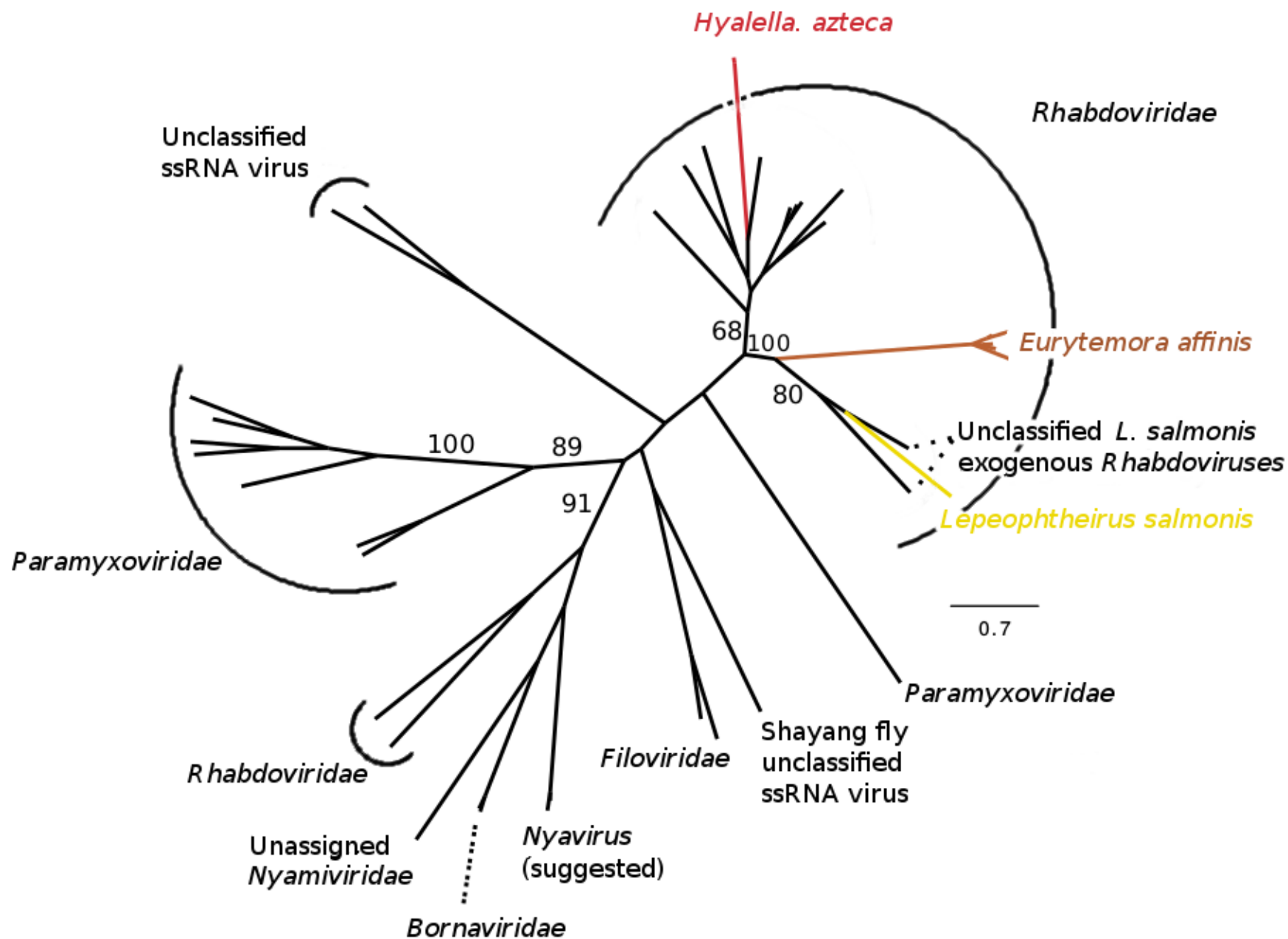

Fig. S3

Supplement: Additional file 6: Figure S3. — Phylogeny of the Mononegavirales group, based on a multiple amino acid alignment and ML analysis of the Mononegavirales-like nucleocapsid protein. In addition to the EVEs discovered in this study, we added sequences of exogenous viruses from the Mononegavirales group. ML nonparametric bootstrap values (100 replicates) are indicated when > 70. (PDF 92 kb) [file 13100_2015_47_MOESM6_ESM.pdf]

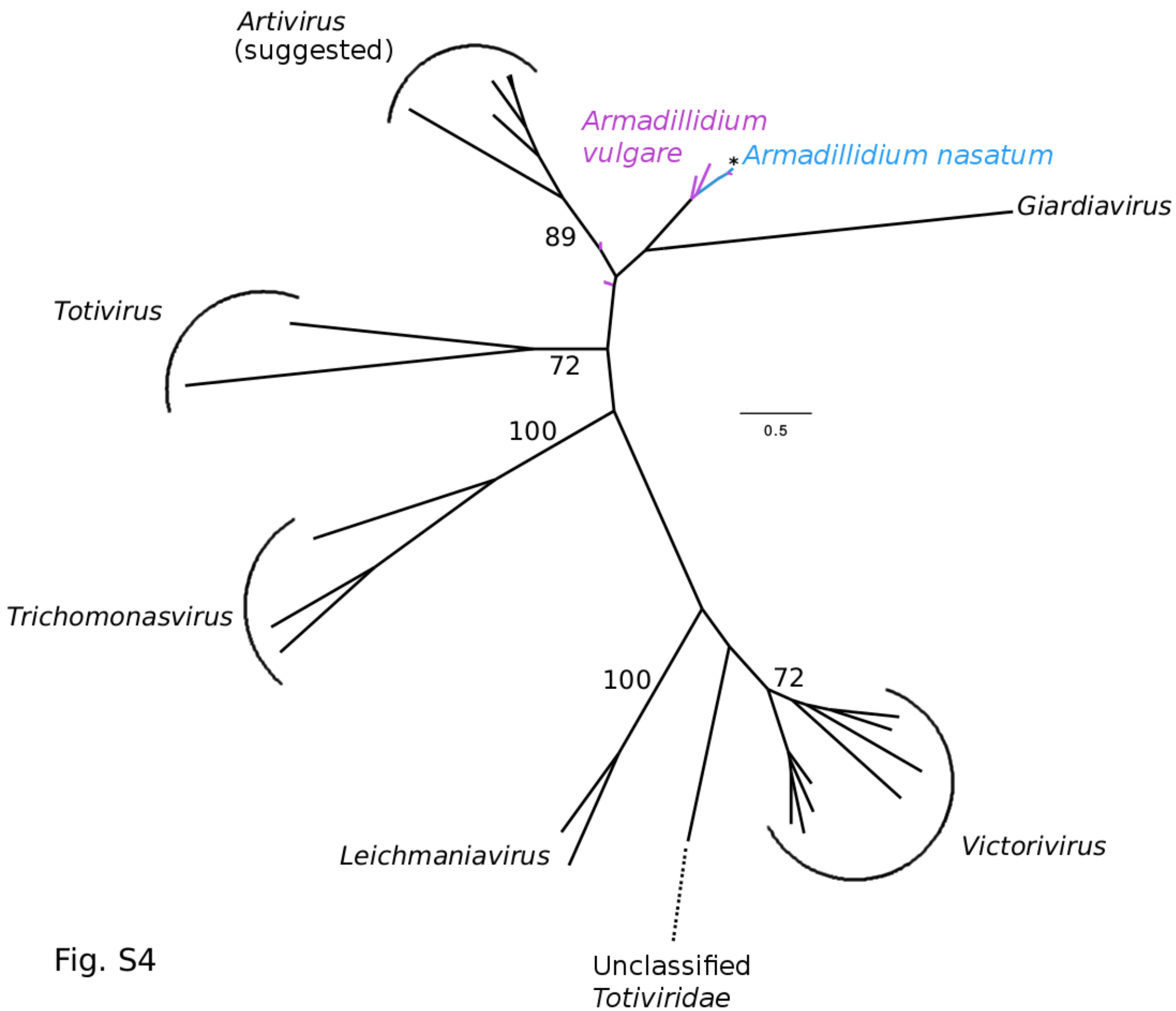

Supplement: Additional file 7: Figure S4. — Phylogeny of the Totiviridae family, based on a multiple amino acid alignment and ML analysis of the nucleocapsid protein. In addition to the EVEs discovered in this study, we added sequences of exogenous viruses from the Totiviridae family. ML nonparametric bootstrap values (100 replicates) are indicated when > 70. (PDF 74 kb) [file 13100_2015_47_MOESM7_ESM.pdf]

a

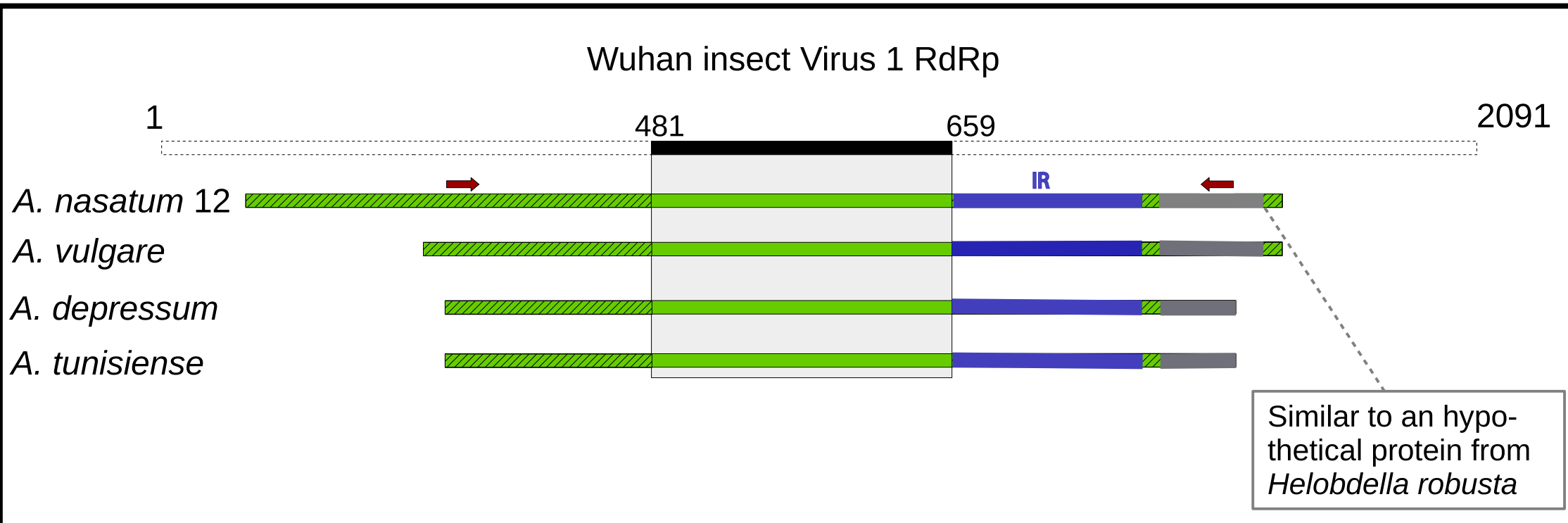

Fig. S5

**b**

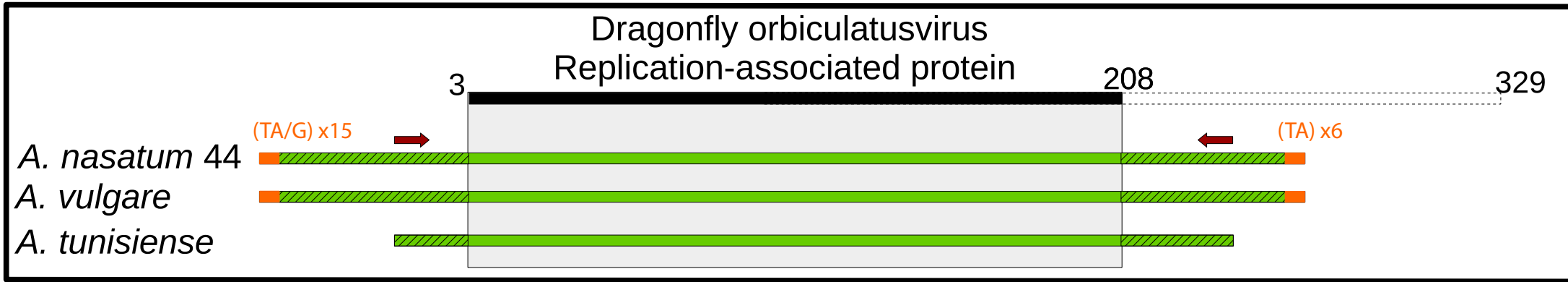

**C**

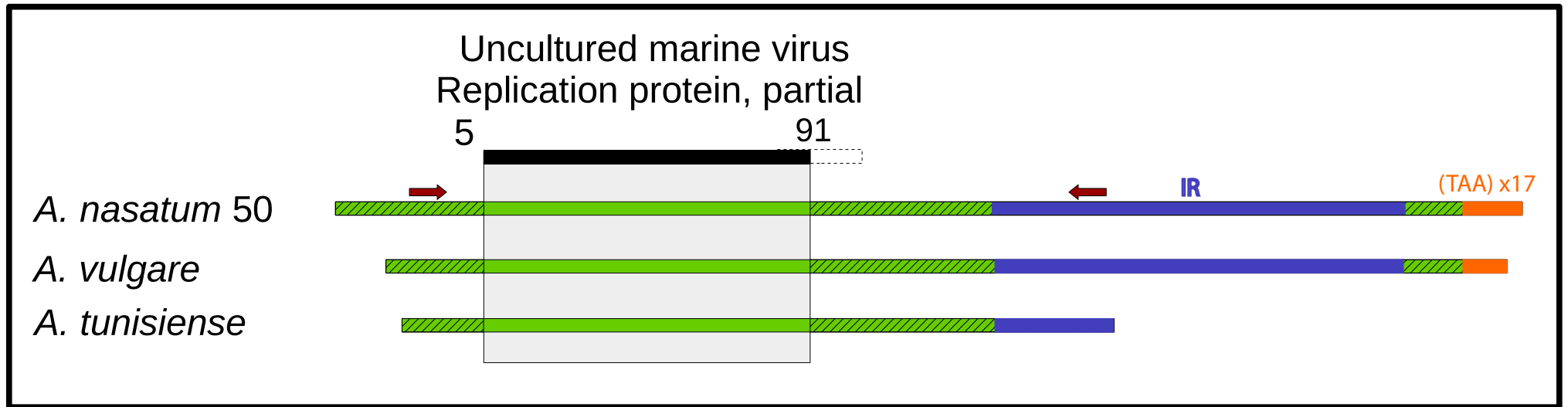

Supplement: Additional file 8: Figure S5. — Schematic representation of the three EVE loci that are orthologous between the various Armadillidium species. The plain green portion of the loci are similar to a virus. a) A. nasatum Bunyavirus-like EVE 12 is most similar to the Wuhan insect virus 1 RdRp (AJG39261). Its 3’ flank contains a 103-bp interspersed repeat (IR in blue) which is repeated at least 66 times in the A. nasatum genome (average similarity between repeats = 86 %) and a partial ORF similar to a hypothetical protein from Helobdella robusta (in grey). b) A. nasatum Circovirus-like EVE 44 is most similar to the Dragonfly orbiculatus virus rep protein (AFS65301). Its 5’ and 3’ flank contain a dinucleotide microsatellite (in orange) repeated at least 15 and 6 times respectively, that are shared at the exact same position with A. vulgare. c) A. nasatum Circovirus-like EVE 50 is most similar to the rep protein of an Uncultured marine virus (GAC77817).xIts 3’ flank contains a 130-bp interspersed repeat which is repeated at least 13 times in the A. nasatum genome (average similarity between repeats = 91 %), as well as a trinucleotide microsatellite repeated at least 17 times and shared with A. vulgare. The green portions of the loci with slanted black stripes correspond to the rest of the flanking regions, which are not similar to any known sequence. Red arrows indicate the position of forward and reverse PCR primers. (PDF 393 kb) [file 13100_2015_47_MOESM8_ESM.pdf]
