# Supplementary material for: Comparative paleovirological analysis of crustaceans identifies multiple widespread viral groups
Source: Mob DNA. 2015 Sep 16;6:16. doi: 10.1186/s13100-015-0047-3 (PMC4573495; doi:10.1186/s13100-015-0047-3)
Supplement: Additional file 3: Table S2. — Characteristics of endogenous viral elements in six crustacean genomes. In the "PCR test" column, hyphens indicate that we did not attempt to amplify the locus by PCR, while the "✓" sign indicates that we successfully amplified the locus by PCR in A. nasatum. The EVEs sharing the same letter (A, B, C, D, E or F) in the "Post insertional duplications" column have identical flanking regions, suggesting they were generated by post insertional duplication. In the same column, hyphens indicate either no flanking region or no similarity of the flanking region to any other EVE locus. (ZIP 139 kb) [file 13100_2015_47_MOESM3_ESM.zip › Table S2.docx]

Table S2 : Characteristics of endogenous viral elements in six crustacean genomes. X: not tested by PCR; ✓ : tested by PCR

| **Species** | **Virus group** | **Order/Family** | **seq nb** | **sequence accession**  **number** | **Contig size (bp)** | **Best blastp hit** | **Protein** | **identies (%)** | **Stop** | **Frame-shift** | **Length of hit (aa)** | **PCR test** |
| --- | --- | --- | --- | --- | --- | --- | --- | --- | --- | --- | --- | --- |
| *Armadillidium nasatum* | -ssRNA | Bunyaviridae | 1 | C30415564 8.0 | 203 | Wuhan Insect virus 1 | RdRp | 63 | 0 | 0 | 56 | X |
|  |  |  | 2 | C32292459 24.0 | 266 | Wuhan Insect virus 1 | RdRp | 67 | 1 | 1 | 42 | X |
|  |  |  | 3 | C34143952 5.0 | 414 | Wuhan Insect virus 1 | RdRp | 51 | 0 | 1 | 137 | X |
|  |  |  | 4 | C34651106 7.0 | 508 | Wuhan Millipede Virus 1 | RdRp | 37 | 4 | 0 | 169 | X |
|  |  |  | 5 | C35122384 7.0 | 697 | Wuhan Millipede Virus 1 | RdRp | 43 | 0 | 1 | 228 | X |
|  |  |  | 6 | C35254382 7.0 | 809 | Wuhan Insect virus 1 | RdRp | 49 | 2 | 2 | 258 | X |
|  |  |  | 7 | scaffold1373 7.1 | 3696 | Wuhan Insect virus 1 | RdRp | 36 | 1 | 1 | 641 | ✓ |
|  |  |  | 8 | scaffold209059 11.4 | 611 | Wuhan Insect virus 1 | RdRp | 45 | 2 | 1 | 186 | X |
|  |  |  | 9 | scaffold225132 11.8 | 603 | Wuhan Insect virus 1 | RdRp | 33 | 1 | 3 | 170 | X |
|  |  |  | 10 | scaffold465514 8.1 | 369 | Wuhan Millipede Virus 1 | RdRp | 43 | 1 | 1 | 104 | X |
|  |  |  | 11 | scaffold508074 3.6 | 905 | Wuhan Insect virus 1 | RdRp | 49 | 0 | 1 | 104 | X |
|  |  |  | 12 | scaffold525835 7.0 | 1443 | Wuhan Millipede Virus 1 | RdRp | 32 | 2 | 1 | 153 | ✓ |
|  |  |  | 13 | C30738472 19.0 | 217 | Wuhan Insect virus 1 | RdRp | 57 | 0 | 1 | 56 | X |
|  |  |  | 14 | scaffold204801 9.5 | 1818 | Whenzhou Shrimp Virus 1 | nucleopasid protein | 40 | 0 | 0 | 141 | X |
|  |  | Mononegavirales | 15 | scaffold200476 7.0 | 2236 | Sanxia Water Strider Virus 4 | RdRp | 25 | 4 | 2 | 593 | ✓ |
|  |  |  | 16 | scaffold727377 12.0 | 1971 | Sanxia Water Strider Virus 4 | RdRp | 27 | 4 | 4 | 425 | ✓ |
|  | dsRNA | Totivirus | 17 | C33386275 29.0 | 334 | Omono River Virus | RdRp | 40 | 0 | 0 | 98 | X |
|  |  |  | 18 | C33735325 35.0 | 365 | Penaeid shrimp infectious myonecrosis virus | RdRp | 30 | 0 | 0 | 113 | X |
|  |  |  | 19 | C35389444 25.0 | 1019 | Tianjin Totivirus | RdRp | 35 | 0 | 0 | 238 | X |
|  |  |  | 20 | C30619484 8.0 | 208 | Penaeid shrimp infectious myonecrosis virus | RdRp | 40 | 0 | 0 | 68 | X |
|  |  |  | 21 | C35524216 11.0 | 1747 | Armigeres subalbatus virus SaX06-AK20 | coat protein | 32 | 0 | 0 | 129 | ✓ |
|  |  |  | 22 | C35298941 12.0 | 862 | Penaeid shrimp infectious myonecrosis virus | RdRp | 36 | 0 | 0 | 94 | X |
|  |  |  | 23 | C35444212 31.0 | 1177 | Penaeid shrimp infectious myonecrosis virus | ORF1/ORF2 polyprotein | 27 | 0 | 0 | 233 | ✓ |
|  |  |  | 24 | scaffold243915 13.5 | 387 | Ixodes scapularis (endogenous) | hypothetical protein IscW_ISCW017951 | 49 | 0 | 0 | 37 | X |
|  | ssDNA | circovirus | 25 | C23786890 31.0 | 110 | Circoviridae 13 LDMD-2013 | replication-associated protein | 56 | 0 | 0 | 36 | X |
|  |  |  | 26 | C26327446 23.0 | 123 | uncultured marine virus | replication protein, partial | 55 | 0 | 0 | 40 | X |
|  |  |  | 27 | C26553422 36.0 | 123 | uncultured marine virus | hypothetical protein | 61 | 0 | 0 | 38 | X |
|  |  |  | 28 | C26707122 7.0 | 123 | uncultured marine virus | replication protein, partial | 54 | 0 | 0 | 41 | X |
|  |  |  | 29 | C26765798 15.0 | 123 | Canary circovirus | replicase | 53 | 0 | 0 | 40 | X |
|  |  |  | 30 | C28221344 32.0 | 150 | Crassostrea gigas (endogenous) | Replication-associated protein | 51 | 0 | 0 | 43 | X |
|  |  |  | 31 | C29187056 2.0 | 176 | uncultured marine virus | hypothetical protein | 54 | 0 | 0 | 54 | X |
|  |  |  | 32 | C29187058 26.0 | 176 | uncultured marine virus | hypothetical protein | 54 | 0 | 0 | 54 | X |
|  |  |  | 33 | C30528525 9.0 | 206 | Crassostrea gigas (endogenous) | Replication-associated protein | 43 | 0 | 0 | 67 | X |
|  |  |  | 34 | C30538511 8.0 | 206 | uncultured marine virus | hypothetical protein | 52 | 0 | 0 | 61 | X |
|  |  |  | 35 | C33301027 19.0 | 327 | uncultured marine virus | hypothetical protein | 57 | 0 | 0 | 67 | X |
|  |  |  | 36 | C33498872 30.0 | 343 | Crassostrea gigas (endogenous) | Replication-associated protein | 41 | 0 | 0 | 66 | X |
|  |  |  | 37 | C33687153 6.0 | 360 | uncultured marine virus | hypothetical protein | 51 | 0 | 0 | 92 | X |
|  |  |  | 38 | C33895310 19.0 | 382 | uncultured marine virus | hypothetical protein | 47 | 0 | 0 | 101 | X |
|  |  |  | 39 | C33895310 19.0 | 402 | uncultured marine virus | hypothetical protein | 44 | 2 | 0 | 93 | X |
|  |  |  | 40 | C34747219 23.0 | 534 | Crassostrea gigas (endogenous) | Replication-associated protein | 41 | 0 | 0 | 81 | X |
|  |  |  | 41 | C34947740 13.0 | 605 | Dragonfly orbiculatusvirus | Replication-associated protein | 38 | 0 | 0 | 198 | X |
|  |  |  | 42 | C35109320 23.0 | 688 | Dragonfly orbiculatusvirus | Replication-associated protein | 49 | 0 | 1 | 114 | X |
|  |  |  | 43 | C35295582 11.0 | 858 | Dragonfly orbiculatusvirus | Replication-associated protein | 33 | 0 | 0 | 212 | X |
|  |  |  | 44 | C35421427 12.0 | 1102 | Dragonfly orbiculatusvirus | Replication-associated protein | 30 | 0 | 1 | 205 | ✓ |
|  |  |  | 45 | C35489999 16.0 | 1408 | Dragonfly orbiculatusvirus | Replication-associated protein | 37 | 1 | 0 | 229 | ✓ |
|  |  |  | 46 | C35551975 21.0 | 2632 | Dragonfly orbiculatusvirus | Replication-associated protein | 36 | 2 | 1 | 257 | ✓ |
|  |  |  | 47 | scaffold187637 7.2 | 1662 | Dragonfly orbiculatusvirus | Replication-associated protein | 44 | 0 | 0 | 81 | X |
|  |  |  | 48 | scaffold199289 10.5 | 616 | uncultured marine virus | replication protein, partial | 51 | 0 | 1 | 76 | X |
|  |  |  | 49 | scaffold506469 4.6 | 927 | Sewage-associated circular DNA virus-21 | Replication-associated protein | 41 | 1 | 0 | 46 | X |
|  |  |  | 50 | scaffold689805 10.6 | 1466 | uncultured marine virus | replication protein, partial | 40 | 1 | 1 | 74 | ✓ |
|  |  |  | 51 | scaffold378771 13.8 | 902 | Dragonfly orbiculatusvirus | Replication-associated protein | 39 | 0 | 0 | 188 | X |
|  |  |  | 52 | scaffold74250 8.0 | 1908 | Dragonfly orbiculatusvirus | Replication-associated protein | 40 | 0 | 0 | 112 | X |
|  |  |  | 53 | scaffold569300 8.3 | 1607 | Dragonfly orbiculatusvirus | Replication-associated protein | 34 | 0 | 0 | 83 | X |
|  |  |  | 54 | C34412062 12.0 | 457 | Dragonfly orbiculatusvirus | Replication-associated protein | 44 | 0 | 0 | 111 | X |
|  |  |  | 55 | C34528419 6.0 | 480 | Dragonfly orbiculatusvirus | Replication-associated protein | 42 | 0 | 0 | 127 | X |
|  |  |  | 56 | scaffold161384 7.1 | 1957 | Dragonfly orbiculatusvirus | Replication-associated protein | 44 | 1 | 0 | 86 | X |
|  | ssDNA | parvovirus | 57 | C25610410 4.0 | 123 | Echinococcus granulosus (endogenous) | Non-capsid protein NS-1 | 48 | 0 | 0 | 40 | X |
|  |  |  | 58 | C25610412 16.0 | 123 | Decapod penstyldensovirus 1 | nonstructural protein 1 | 53 | 0 | 0 | 40 | X |
|  |  |  | 59 | C26581986 8.0 | 123 | Penaeus stylirostris densovirus | NS1-like protein 2 | 53 | 0 | 0 | 40 | X |
|  |  |  | 60 | C27523145 | 135 | Penaeus stylirostris densovirus | NS1-like protein 2 | 53 | 0 | 0 | 43 | X |
|  |  |  | 61 | C28301606 8.0 | 152 | Penaeus stylirostris densovirus | NS1-like protein 2 | 51 | 0 | 0 | 43 | X |
|  |  |  | 62 | C28345975 6.0 | 153 | Decapod penstyldensovirus 1 | noncapsid protein NS1, partial | 45 | 0 | 0 | 51 | X |
|  |  |  | 63 | C28345977 5.0 | 153 | Decapod penstyldensovirus 1 | noncapsid protein NS1, partial | 43 | 0 | 0 | 51 | X |
|  |  |  | 64 | C31918795 14.0 | 250 | Decapod penstyldensovirus 1 | NS1 | 47 | 0 | 0 | 75 | X |
|  |  |  | 65 | C31966103 23.0 | 252 | Decapod penstyldensovirus 1 | noncapsid protein NS1, partial | 46 | 0 | 0 | 81 | X |
|  |  |  | 66 | C34415820 6.0 | 458 | Decapod penstyldensovirus 1 | NS1 | 31 | 0 | 0 | 153 | X |
|  |  |  | 67 | C35540421 16.0 | 2088 | Penaeus monodon (endogenous) | non-structural protein 1 | 30 | 0 | 2 | 389 | ✓ |
|  |  |  | 68 | scaffold557885 4.6 | 511 | Penaeus monodon (endogenous) | non-structural protein 1 | 32 | 1 | 0 | 130 | X |
|  |  |  | 69 | scaffold639266 8.8 | 572 | Penaeus stylirostris densovirus | NS1-like proten 1 | 38 | 3 | 0 | 37 | ✓ |
| **Species** | **Virus group** | **Order/Family** | **seq nb** | **sequence accession** | **Contig size (bp)** | **Best blastp hit** | **Protein^$^** | **identies (%)** | **Stop** | **Frameshift** | **Length of hit (aa)** | **PCR test** |
| *Daphnia pulex* | -ssRNA | Bunyaviridae | 1 | ACJG01017696* | 1221 | Whenzhou Shrimp Virus 1 | RdRp | 45 | 1 | 0 | 71 | X |
|  |  |  | 2 | ACJG01015620 | 3707 | Changping Tick Virus 1 | RdRp | 39 | 2 | 2 | 276 | X |
|  |  |  | 3 | ACJG01015074 | 1178 | Silverwater virus | RdRp | 35 | 0 | 0 | 114 | X |
|  |  |  | 4 | ACJG01009400 | 1694 | Punta Toro virus | L protein | 46 | 1 | 1 | 138 | X |
|  |  |  | 5 | ACJG01007846* | 1672 | Chize virus | RNA polymerase | 51 | 0 | 0 | 177 | X |
|  |  |  | 6 | ACJG01006471 | 2078 | Silverwater virus | RdRp | 37 | 2 | 0 | 100 | X |
|  |  |  | 7 | ACJG01006381 | 97052 | Manawa virus | polymerase | 40 | 10 | 3 | 591 | X |
|  |  |  | 8 | ACJG01005250 | 19632 | EgAN 1825-61 virus | polymerase | 26 | 2 | 2 | 290 | X |
|  |  |  | 9 | ACJG01004899 | 56569 | Silverwater virus | RdRp | 41 | 12 | 3 | 955 | X |
|  |  |  | 10 | ACJG01004740 | 70739 | Blacklegged tick phlebovirus-1 | L protein | 30 | 15 | 2 | 867 | X |
|  |  |  | 11 | ACJG01003328 | 39731 | Rift Valley fever virus | polymerase | 34 | 7 | 4 | 860 | X |
|  |  |  | 12 | ACJG01002369* | 8945 | Murre virus | RNA polymerase | 44 | 0 | 0 | 219 | X |
|  |  |  | 13 | ACJG01002368 | 25791 | Blacklegged tick phlebovirus-1 | L protein | 41 | 0 | 0 | 492 | X |
|  |  |  | 14 | ACJG01002210 | 70728 | Manawa virus | polymerase | 37 | 9 | 2 | 426 | X |
|  |  |  | 15 | ACJG01001622 | 81044 | Murre virus | RNA polymerase | 50 | 1 | 2 | 546 | X |
|  |  |  | 16 | ACJG01001614 | 64754 | EgAN 1825-61 virus | polymerase | 42 | 3 | 2 | 297 | X |
|  |  |  | 17 | ACJG01001533 | 44407 | Blacklegged tick phlebovirus-2 | L protein | 35 | 12 | 2 | 803 | X |
|  |  |  | 18 | ACJG01001371 | 36808 | RML-105355 virus | RNA polymerase | 36 | 9 | 1 | 520 | X |
|  |  |  | 19 | ACJG01000925 | 61325 | Arumowot virus | polymerase | 33 | 6 | 1 | 353 | X |
|  | ssDNA | parvovirus | 20 | ACJG01006381 | 97052 | Daphnia pulex (endogenous) | hypothetical protein DAPPUDRAFT_331456 | 100 | 0 | 0 | 43 | X |
|  |  |  | 21 | ACJG01001970 | 21120 | Daphnia pulex (endogenous) | hypothetical protein DAPPUDRAFT_331456 | 100 | 0 | 0 | 53 | X |
|  |  |  | 22 | ACJG01001314 | 105517 | Daphnia pulex (endogenous) | hypothetical protein DAPPUDRAFT_331456 | 100 | 0 | 1 | 166 | X |
| **Species** | **Virus group** | **Order/Family** | **seq nb** | **sequence accession** | **Contig size (bp)** | **Best blastp hit** | **Protein^$^** | **identies (%)** | **Stop** | **Frameshift** | **Length of hit (aa)** | **PCR test** |
| *Daphnia pulicaria* | -ssRNA | Bunyaviridae | 1 | ANIS4191.x1 | 486 | Manawa virus | polymerase, partial | 45 | 2 | 1 | 129 | X |
|  |  |  | 2 | ANIS948.x1 | 600 | Yongjia Tick Virus 1 | RdRp | 42 | 0 | 1 | 104 | X |
|  |  |  | 3 | ANIS13900.y2 | 1088 | Munguba virus | polymerase | 45 | 1 | 3 | 147 | X |
|  |  |  | 4 | ANIS15345.x2 | 1061 | Precarious point virus | polymerase | 26 | 11 | 2 | 403 | X |
|  |  |  | 5 | ANIS18905.x2 | 1115 | Khasan virus | RdRp | 55 | 0 | 0 | 92 | X |
|  |  |  | 6 | ANIS18938.y2 | 1066 | Grand Arbaud virus | RNA polymerase | 56 | 1 | 0 | 52 | X |
|  |  |  | 7 | ANIS19419.y2 | 1107 | Silverwater virus | RdRp | 40 | 4 | 1 | 281 | X |
|  |  |  | 8 | ANIS76304.y1 | 882 | RML-105355 virus | RNA polymerase | 46 | 2 | 0 | 184 | X |
|  |  |  | 9 | ANIS116485.x1 | 683 | Toscana virus | polymerase | 45 | 2 | 1 | 206 | X |
|  |  |  | 10 | ANIS147191.y1 | 873 | Dabieshan Tick Virus | RdRp | 46 | 5 | 1 | 85 | X |
|  |  |  | 11 | ANIS151895.y1 | 900 | Dabieshan Tick Virus | RdRp | 46 | 5 | 1 | 85 | X |
|  |  |  | 12 | ANIS158738.g1 | 980 | RML-105355 virus | RNA polymerase | 44 | 7 | 1 | 245 | X |
|  |  |  | 13 | ANIS163603.b1 | 1022 | Grand Arbaud virus | RNA polymerase | 66 | 10 | 2 | 283 | X |
|  |  |  | 14 | ANIS164171.b1 | 970 | RML-105355 virus | RNA polymerase | 45 | 7 | 1 | 255 | X |
|  |  |  | 15 | ANIS165444.y3 | 757 | Blacklegged tick phlebovirus-2 | L protein | 46 | 5 | 1 | 235 | X |
|  |  |  | 16 | ANIS166873.g1 | 975 | Severe fever with thrombocytopenia syndrome virus | RNA polymerase | 47 | 2 | 3 | 154 | X |
|  |  |  | 17 | ANIS173772.g1 | 950 | RML-105355 virus | RNA polymerase | 38 | 7 | 0 | 259 | X |
|  |  |  | 18 | ANIS174187.b1 | 1017 | Huangpi Tick Virus 2 | RdRp | 39 | 9 | 2 | 208 | X |
|  |  |  | 19 | ANIS194711.b1 | 966 | Munguba virus | polymerase | 41 | 3 | 1 | 281 | X |
|  |  |  | 20 | ANIS199857.g1 | 1017 | Uukuniemi virus | RdRp | 45 | 4 | 2 | 262 | X |
|  |  |  | 21 | ANIS219560.x2 | 1075 | Lihan Tick Virus | RdRp | 44 | 2 | 1 | 96 | X |
|  |  |  | 22 | ANIS221787.x1 | 1002 | Toscana virus | RdRp | 40 | 0 | 0 | 47 | X |
|  |  |  | 23 | ANIS223258.y3 | 617 | Blacklegged tick phlebovirus-2 | L protein | 41 | 0 | 3 | 165 | X |
|  |  |  | 24 | ANIS224538.x2 | 1147 | Grand Arbaud virus | RNA polymerase | 42 | 0 | 0 | 78 | X |
|  |  |  | 25 | ANIS225848.y1 | 781 | Lihan Tick Virus | RdRp | 46 | 1 | 0 | 140 | X |
|  |  |  | 26 | ANIS226979.y2 | 1112 | Phlebovirus GGP-2011a | polymerase | 24 | 2 | 1 | 291 | X |
|  |  |  | 27 | ANIS228021.x1 | 673 | Punta Toro virus | L protein | 39 | 6 | 0 | 174 | X |
|  |  |  | 28 | ANIS231044.g1 | 973 | Blacklegged tick phlebovirus-2 | L protein | 35 | 8 | 2 | 333 | X |
|  |  |  | 29 | ANIS231965.b1 | 926 | RML-105355 virus | RNA polymerase | 41 | 2 | 0 | 201 | X |
|  |  |  | 30 | ANIS232409.g1 | 960 | Lihan Tick Virus | RdRp | 37 | 2 | 1 | 162 | X |
|  |  |  | 31 | ANIS232957.g1 | 1008 | Murre virus | RNA polymerase | 41 | 1 | 1 | 211 | X |
|  |  |  | 32 | ANIS233368.b1 | 892 | Manawa virus | polymerase, partial | 33 | 6 | 2 | 383 | X |
|  |  |  | 33 | ANIS234222.g1 | 997 | Blacklegged tick phlebovirus-2 | L protein | 40 | 1 | 1 | 306 | X |
|  |  |  | 34 | ANIS240533.b1 | 971 | Dabieshan Tick Virus | RdRp | 44 | 8 | 1 | 236 | X |
|  |  |  | 35 | ANIS242341.b1 | 978 | Komandory virus | RdRp | 40 | 2 | 1 | 150 | X |
|  |  |  | 36 | ANIS248221.y1 | 691 | Bole Tick Virus 1 | RdRp | 43 | 3 | 0 | 138 | X |
|  |  |  | 37 | ANIS248927.y1 | 990 | Yongjia Tick Virus 1 | RdRp | 34 | 1 | 2 | 242 | X |
|  |  |  | 38 | ANIS250506.x1 | 834 | Khasan virus | RdRp | 38 | 1 | 1 | 244 | X |
|  |  |  | 39 | ANIT2005.g2 | 932 | Blacklegged tick phlebovirus-1 | L protein | 47 | 7 | 1 | 253 | X |
|  |  |  | 40 | ANIT3592.g2 | 1023 | Dabieshan Tick Virus | RdRp | 48 | 3 | 1 | 140 | X |
|  |  |  | 41 | ANIT80995.b1 | 1045 | RML-105355 virus | RNA polymerase | 67 | 6 | 3 | 240 | X |
|  |  |  | 42 | ANIT81019.g1 | 983 | Grand Arbaud virus | RNA polymerase | 42 | 0 | 1 | 78 | X |
|  |  |  | 43 | ANIT81587.g1 | 1018 | RML-105355 virus | RNA polymerase | 44 | 1 | 1 | 325 | X |
|  |  |  | 44 | ANIT81628.g1 | 1036 | Kaisodi virus | RdRp | 40 | 3 | 1 | 86 | X |
|  |  |  | 45 | ANIT85177.g1 | 1016 | American dog tick phlebovirus | L protein | 45 | 3 | 3 | 283 | X |
|  |  |  | 46 | ANIT86855.g1 | 1013 | Sandfly fever Sicilian virus | RdRp | 29 | 2 | 2 | 261 | X |
|  |  |  | 47 | ANIT87096.g1 | 980 | Whenzhou Shrimp Virus 1 | RdRp | 73 | 1 | 0 | 67 | X |
|  |  |  | 48 | ANIT87409.g1 | 986 | Severe fever with thrombocytopenia syndrome virus | RNA polymerase | 43 | 1 | 0 | 61 | X |
|  |  |  | 49 | ANIT103731.x2 | 1083 | Lihan Tick Virus | RdRp | 44 | 2 | 1 | 96 | X |
|  |  |  | 50 | ANIT105923.y2 | 1053 | Punta Toro virus | L protein | 44 | 3 | 2 | 288 | X |
|  |  |  | 51 | ANIT105984.y2 | 1122 | Dabieshan Tick Virus | RdRp | 54 | 0 | 1 | 114 | X |
|  |  |  | 52 | ANIT107263.y2 | 1049 | Blacklegged tick phlebovirus-1 | L protein | 40 | 10 | 0 | 270 | X |
|  |  |  | 53 | ANIT108444.x2 | 1075 | Lihan Tick Virus | RdRp | 41 | 8 | 2 | 336 | X |
|  |  |  | 54 | ANIT124483.x2 | 1094 | Grand Arbaud virus | RNA polymerase | 49 | 1 | 1 | 223 | X |
|  |  |  | 55 | ANIT145147.g1 | 950 | Manawa virus | polymerase, partial | 41 | 2 | 1 | 146 | X |
|  |  |  | 56 | ANIT160860.g1 | 824 | RML-105355 virus | RNA polymerase | 45 | 1 | 0 | 91 | X |
|  |  |  | 57 | ANIT161061.b1 | 1021 | Zaliv Terpenia virus | polymerase | 42 | 0 | 1 | 244 | X |
|  |  |  | 58 | ANIT161307.b1 | 976 | Yongjia Tick Virus 1 | RdRp | 34 | 1 | 1 | 219 | X |
|  |  |  | 59 | ANIT162598.g1 | 1016 | Komandory virus | RdRp | 44 | 2 | 2 | 177 | X |
|  |  |  | 60 | ANIT170161.g1 | 976 | Huangpi Tick Virus 2 | RdRp | 69 | 4 | 3 | 212 | X |
|  |  |  | 61 | ANIT171709.g1 | 990 | Dabieshan Tick Virus | RdRp | 42 | 2 | 0 | 151 | X |
|  |  |  | 62 | ANIT193202.g1 | 1014 | Zaliv Terpenia virus | RdRp | 32 | 6 | 2 | 328 | X |
|  |  |  | 63 | ANIT194018.g1 | 957 | Whenzhou Shrimp Virus 1 | RdRp | 43 | 1 | 0 | 67 | X |
|  |  |  | 64 | ANIT205129.b1 | 963 | Manawa virus | polymerase, partial | 70 | 3 | 2 | 241 | X |
|  |  |  | 65 | ANIT206008.b1 | 983 | Arumowot virus | polymerase | 43 | 8 | 2 | 327 | X |
|  |  |  | 66 | ANIT207425.g1 | 1001 | Khasan virus | RdRp | 69 | 2 | 3 | 213 | X |
|  |  |  | 67 | ANIT207704.b1 | 1021 | Cocle virus | L protein | 45 | 2 | 1 | 192 | X |
|  |  |  | 68 | ANIT233174.b1 | 928 | Dabieshan Tick Virus | RdRp | 46 | 3 | 1 | 165 | X |
|  |  |  | 69 | ANIT234502.g1 | 940 | Huangpi Tick Virus 2 | RdRp | 40 | 1 | 2 | 160 | X |
|  | ssDNA | Circoviridae | 71 | ANIT146708.b1 | 1033 | Daphnia pulex (endogenous) | hypothetical protein DAPPUDRAFT_328803 | 57 | 1 | 0 | 174 | X |
|  |  |  | 72 | ANIT159840.b1 | 928 | Daphnia pulex (endogenous) | hypothetical protein DAPPUDRAFT_328803 | 60 | 0 | 0 | 187 | X |
|  |  |  | 73 | ANIT228531.b1 | 1020 | Daphnia pulex (endogenous) | hypothetical protein DAPPUDRAFT_328803 | 59 | 1 | 0 | 179 | X |
|  | ssDNA | Parvoviridae | 74 | ANIS196645.b1 | 974 | Daphnia pulex (endogenous) | hypothetical protein DAPPUDRAFT_331456 | 87 | 0 | 0 | 54 | X |
|  |  |  | 75 | ANIS238741.g1 | 931 | Daphnia pulex (endogenous) | hypothetical protein DAPPUDRAFT_331456 | 83 | 2 | 1 | 100 | X |
| **Species** | **Virus group** | **Order/Family** | **seq nb** | **sequence accession** | **Contig size (bp)** | **Best blastp hit** | **Protein^$^** | **identies (%)** | **Stop** | **Frameshift** | **Length of hit (aa)** | **PCR test** |
| *Eurytemora affinis* | -ssRNA | Mononegavirales | 1 | AZAI01098724 | 43001 | Maraba virus | RdRp | 31 | 2 | 4 | 1099 | X |
|  |  |  | 2 | AZAI01080697 | 12042 | Vesicular stomatitis Alagoas virus | L polymerase protein | 30 | 4 | 2 | 1257 | X |
|  |  |  | 3 | AZAI01122312 | 5714 | Lepeophtheirus salmonis rhabdovirus No127 | nucleoprotein | 34 | 0 | 0 | 148 | X |
|  |  |  | 4 | AZAI01116005 | 8441 | Kern Canyon virus | polymerase | 29 | 10 | 4 | 825 | X |
|  |  |  | 5 | AZAI01090152 | 7951 | Le Dantec virus | nucleoprotein | 26 | 0 | 0 | 301 | X |
|  |  |  | 6 | AZAI01084048 | 17155 | Chandipura virus IB An 9978 | nucleocapsid protein | 27 | 0 | 0 | 199 | X |
|  |  |  | 7 | AZAI01069314 | 2825 | Chandipura virus IB An 9978 | nucleocapsid protein | 26 | 0 | 0 | 200 | X |
|  |  |  | 8 | AZAI01056871 | 4673 | Sweetwater Branch virus | nucleoprotein | 26 | 1 | 0 | 140 | X |
|  |  |  | 9 | AZAI01020236* | 8070 | Chandipura virus IB An 9978 | Nucleocapsid protein | 29 | 0 | 0 | 198 | X |
|  |  |  | 10 | AZAI01009144 | 4715 | Tibrogargan virus | nucleoprotein N | 25 | 0 | 0 | 263 | X |
| **Species** | **Virus group** | **Order/Family** | **seq nb** | **sequence accession** | **Contig size (bp)** | **Best blastp hit** | **Protein^$^** | **identies (%)** | **Stop** | **Frameshift** | **Length of hit (aa)** | **PCR test** |
| *Hyalella azteca* | -ssRNA | Mononegavirales | 1 | JQDR01191785 | 21369 | Wenzhou Crab Virus 1 | RdRp | 38 | 6 | 4 | 1071 | X |
|  |  |  | 2 | JQDR01149589 | 21313 | Wenzhou Crab Virus 1 | RdRp | 41 | 2 | 1 | 889 | X |
|  |  |  | 3 | JQDR01142712 | 9075 | Wenzhou Crab Virus 1 | RdRp | 44 | 10 | 4 | 922 | X |
|  |  |  | 4 | JQDR01096877 | 3288 | Wenzhou Crab Virus 1 | RdRp | 46 | 0 | 1 | 976 | X |
|  |  |  | 5 | JQDR01090109 | 6575 | Wenzhou Crab Virus 1 | RdRp | 30 | 9 | 6 | 1059 | X |
|  |  |  | 6 | JQDR01090108 | 11031 | Wenzhou Crab Virus 1 | RdRp | 49 | 2 | 5 | 1041 | X |
|  |  |  | 7 | JQDR01079660 | 17686 | Wenzhou Crab Virus 1 | RdRp | 48 | 7 | 4 | 1326 | X |
|  |  |  | 8 | JQDR01078020 | 16871 | Wenzhou Crab Virus 1 | RdRp | 43 | 3 | 4 | 711 | X |
|  |  |  | 9 | JQDR01069010 | 7841 | Wenzhou Crab Virus 1 | RdRp | 32 | 0 | 3 | 1150 | X |
|  |  |  | 10 | JQDR01062028 | 43442 | Wenzhou Crab Virus 1 | RdRp | 37 | 13 | 4 | 1215 | X |
|  |  |  | 11 | JQDR01055259 | 16006 | Wenzhou Crab Virus 1 | RdRp | 46 | 0 | 3 | 888 | X |
|  |  |  | 12 | JQDR01047157 | 4560 | Wenzhou Crab Virus 1 | RdRp | 37 | 1 | 0 | 1327 | X |
|  |  |  | 13 | JQDR01044883 | 1911 | Walkabout Creek virus | nucleoprotein | 28 | 0 | 0 | 140 | X |
|  |  |  | 14 | JQDR01042035 | 2452 | Walkabout Creek virus | nucleoprotein | 27 | 0 | 0 | 370 | X |
|  |  |  | 15 | JQDR01038354 | 7146 | Wenzhou Crab Virus 1 | RdRp | 36 | 6 | 0 | 1601 | X |
|  |  |  | 16 | JQDR01038328 | 14700 | Wenzhou Crab Virus 1 | RdRp | 44 | 6 | 4 | 870 | X |
|  |  |  | 17 | JQDR01027418 | 16055 | Wenzhou Crab Virus 1 | RdRp | 51 | 7 | 2 | 995 | X |
|  |  |  | 18 | JQDR01014642 | 13652 | Wenzhou Crab Virus 1 | RdRp | 39 | 3 | 5 | 1190 | X |
|  |  |  | 19 | JQDR01044883 | 1911 | Walkabout Creek virus | nucleoprotein | 28 | 0 | 0 | 140 | X |
|  | ssDNA | Circoviridae | 20 | JQDR01129029 | 21973 | Dragonfly orbiculatusvirus | Replication-associated protein | 34 | 4 | 0 | 302 | X |
|  |  |  | 21 | JQDR01075335 | 1824 | Dragonfly orbiculatusvirus | Replication-associated protein | 34 | 4 | 0 | 301 | X |
|  |  |  | 22 | JQDR01037320 | 12836 | Dragonfly orbiculatusvirus | Replication-associated protein | 38 | 0 | 1 | 269 | X |
| **Species** | **Virus group** | **Order/Family** | **seq nb** | **sequence accession** | **Contig size (bp)** | **Best blastp hit** | **Protein^$^** | **identies (%)** | **Stop** | **Frameshift** | **Length of hit (aa)** | **PCR test** |
| Lepeophtheirus *salmonis* | -ssRNA | Bunyaviridae | 1 | ADND02041943 | 6991 | Wuhan Fly Virus 1 | nucleopasid protein | 31 | 1 | 0 | 402 | X |
|  | ssDNA | Circoviridae | 2 | ADND02114629 | 1052 | Bat circovirus | Rep | 42 | 0 | 0 | 103 | X |
|  |  |  | 3 | ADND02041958* | 6191 | uncultured Nanovirus | Rep | 34 | 1 | 0 | 173 | X |
|  |  |  | 4 | ADND02016799* | 10467 | Raven circovirus | Rep | 36 | 0 | 0 | 142 | X |
|  |  |  | 5 | ADND02012141 | 16909 | Raven circovirus | Rep | 74 | 0 | 0 | 69 | X |
|  |  |  | 6 | ADND02011981 | 12760 | Avon-Heathcote Estuary associated circular virus 24 (endogenous) | replication-associated protein | 61 | 1 | 2 | 237 | X |
|  |  |  | 7 | ADND02001485 | 21783 | uncultured marine virus | hypothetical protein | 29 | 0 | 1 | 252 | X |
|  |  |  | 8 | ADND02087360 | 6106 | uncultured marine virus | hypothetical protein | 37 | 0 | 0 | 115 | X |
|  |  |  | 9 | ADND02051930 | 8256 | McMurdo Ice Shelf pond-associated circular DNA virus-1 (endogenous) | replication-associated protein | 30 | 0 | 0 | 229 | X |
|  | -ssRNA | Mononegavirales | 10 | ADND02004053 | 30907 | Lepeophtheirus salmonis rhabdovirus No127 | nucleoprotein | 31 | 8 | 0 | 311 | X |
|  | ssDNA | Parvoviridae | 11 | ADND02080325 | 3610 | Penaeus monodon (endogenous) | non-structural protein 1 | 28 | 0 | 1 | 382 | X |
|  |  |  | 12 | ADND02047016 | 8405 | Penaeus monodon (endogenous) | non-structural protein 1 | 29 | 0 | 0 | 255 | X |
|  |  |  | 13 | ADND02026560 | 12345 | Penaeus monodon (endogenous) | non-structural protein 1 | 30 | 0 | 0 | 255 | X |
