# Supplementary material for: Comparative paleovirological analysis of crustaceans identifies multiple widespread viral groups
Source: Mob DNA. 2015 Sep 16;6:16. doi: 10.1186/s13100-015-0047-3 (PMC4573495; doi:10.1186/s13100-015-0047-3)
Supplement: Additional file 4: Table S1. — Quality assessments of the 6 crustacean genomes used in this study. (DOCX 17 kb) [file 13100_2015_47_MOESM4_ESM.docx]

**Table S1: Quality assessments of the 6 crustacean genomes used in this study.**

| Species | Estimated genome size (pg)* | Genome assembly size (Gb)** | Number of contigs / scaffolds | N50*** |
| --- | --- | --- | --- | --- |
| *A. vulgare* | *1.96* | *1.5* | *5348537* | *468* |
| *A. nasatum* | NA | 1.5 | 5056750 | 463 |
| *D. pulex* | 0.23 – 0.37 | 0.197 | 18987 | 49267 |
| *D. pulicaria* | 0.24 – 0.49 | 0.317 | 317062 | 981 |
| *H. azteca* | NA | 1.179 | 215630 | 5445 |
| *E. affinis* | 0.63 | 0.495 | 122625 | 5738 |
| *L. salmonis* | 0.58 | 0.790 | 149463 | 9736 |

***Estimated genome sizes (in pg) are coming from the Animal Genome Size Database (**[**http://www.genomesize.com**](http://www.genomesize.com)**).**

****Genome assembly size (in Gb) represents the number of base in the fasta files.**

*****The N50 (in base pairs) corresponds to the length for which all contigs of that length or longer contain at least 50% of the total assembled base pairs.**

**NA: not available.**

**A. vulgare statistics are taken from Thézé et al. (2014).**
