# Supplementary material for: Comparative paleovirological analysis of crustaceans identifies multiple widespread viral groups
Source: Mob DNA. 2015 Sep 16;6:16. doi: 10.1186/s13100-015-0047-3 (PMC4573495; doi:10.1186/s13100-015-0047-3)
Supplement: Additional file 9: Table S3. — PCR primers used to confirm the presence of EVEs uncovered by the bio-informatic analysis and to screen for orthologous insertions in 3 Armadillidae species : Armadillidium vulgare, A. tunisiense and A. depressum. (DOCX 17 kb) [file 13100_2015_47_MOESM9_ESM.docx]

**Table S3 :** PCR primers used to conﬁrm the presence of EVEs uncovered by the bio-informatic analysis and to screen for orthologous insertions in 3 Armadillidae species : *Armadillidium* *vulgare*, *A*. *tunisiense* and *A*. *depressum*

| **Forward Primer** | | | **Reverse Primer** | | **Expected size (pb)** |
| --- | --- | --- | --- | --- | --- |
| **EVE *id*** | **Sequence** | **Tm (°C)** | **Sequence** | **Tm (°C)** |  |
| *A. nasatum* 7 | TGGGATTGTGCATCATCATCAGC | 57.8 | GATTTTTAACCTA  ACGACACCGTTG | 54.6 | 260 |
| *A. nasatum* 12 | GAGAGAATTCCATGCGTGTTACG | 56 | CAAGTGATCTAC  CTTATGCCTGC | 55.5 | 1105 |
| *A. nasatum* 15 | CTGGGAAAAAATAGTATTAGAAGAAAATG | 51.4 | ATGTTGAATTAGG  GTCCGTTTG | 53.3 | 228 |
| *A. nasatum* 16 | CTCCTCTACCCAGCAACAGC | 57.7 | AGGGAGTGAGAG  AGAGGCTAC | 57.3 | 689 |
| *A. nasatum* 21 | AATCACTTTACCGTTTGAGCCAC | 55.8 | GATAGGTGATAA  GGCGTGGAGTC | 56.7 | 854 |
| *A. nasatum* 23 | TGATAACCTTCCTGACTGTCTTCG | 56.2 | CCGTATGTTGG  CAGTAATCCG | 55.8 | 678 |
| *A. nasatum* 44 | ACAATAACAGACTAAGGTCCCTC | 53.8 | AAGTTTCAGCA  GAATTGGCTG | 53.9 | 870 |
| *A. nasatum* 45 | CATTGCCCTAT  TCGAGAAGAG | 53 | GAGTGGGCAGA  ACCAAATCG | 56.3 | 466 |
| *A. nasatum* 46 | CTTCAATACCA  CCTCACTCTGC | 53.3 | CAATGCCTGTG  AACTTTGGC | 55.1 | 818 |
| *A. nasatum* 50 | GAGCTTTACTACCACTTCACTCTG | 54.9 | GCACTCCTTTCGT  AGCCTCACTG | 56.4 | 957 |
| *A. nasatum* 67 | GATTTTTATCCTGT  CTGTCTGTCTC | 53.3 | CTTCCAACGTTTC  GAACATCATC | 54.4 | 540 |
| *A. nasatum* 69 | CCATCCTGTCT  CAGTTACTTTC | 52.8 | CGATACCTCTAGA  ATTTTCCGATAC | 52.7 | 866 |
